# Construction of High-Performance Membranes for Vanadium Redox Flow Batteries: Challenges, Development, and Perspectives

**DOI:** 10.1007/s40820-025-01736-x

**Published:** 2025-05-19

**Authors:** Tan Trung Kien Huynh, Tong Yang, Nayanthara P S, Yang Yang, Jiaye Ye, Hongxia Wang

**Affiliations:** 1 School of Chemistry and Physics, Faculty of Science, Queensland University of Technology, 2 George Street, Brisbane, QLD 4000 Australia; 2 Centre for Materials Science, Queensland University of Technology, 2 George Street, Brisbane, QLD 4000 Australia

**Keywords:** Vanadium redox flow batteries, Membrane, Energy storage

## Abstract

While being a promising candidate for large-scale energy storage, the current market penetration of vanadium redox flow batteries (VRFBs) is still limited by several challenges. As one of the key components in VRFBs, a membrane is employed to separate the catholyte and anolyte to prevent the vanadium ions from cross-mixing while allowing the proton conduction to maintain charge balance in the system during operation. To overcome the weakness of commercial membranes, various types of membranes, ranging from ion exchange membranes with diverse functional groups to non-ionic porous membranes, have been designed and reported to achieve higher ionic conductivity while maintaining low vanadium ion permeability, thus enhancing efficiency. In addition, besides overall efficiency, stability and cost-effectiveness of the membrane are also critical aspects that determine the practical applicability of the membranes and thus VRFBs. In this article, we have offered comprehensive insights into the mechanism of ion transportation in membranes of VRFBs that contribute to the challenges and issues of VRFB applications. We have further discussed optimal strategies for solving the trade-off between the membrane efficiency and its durability in VRFB applications. The development of state-of-the-art membranes through various material and structure engineering is demonstrated to reveal the relationship of properties-structure-performance.

## Highlights

- Critically analyses the ion transport mechanisms of various membranes and compares them and highlights the challenges of membranes for vanadium redox flow battery (VRFB).
- In-depth analysis and discussion of the best strategies for membranes to achieve high-performance VRFB.
- Prospective approaches to realising high-performance, sustainable VRFB membranes.

## Graphical abstract

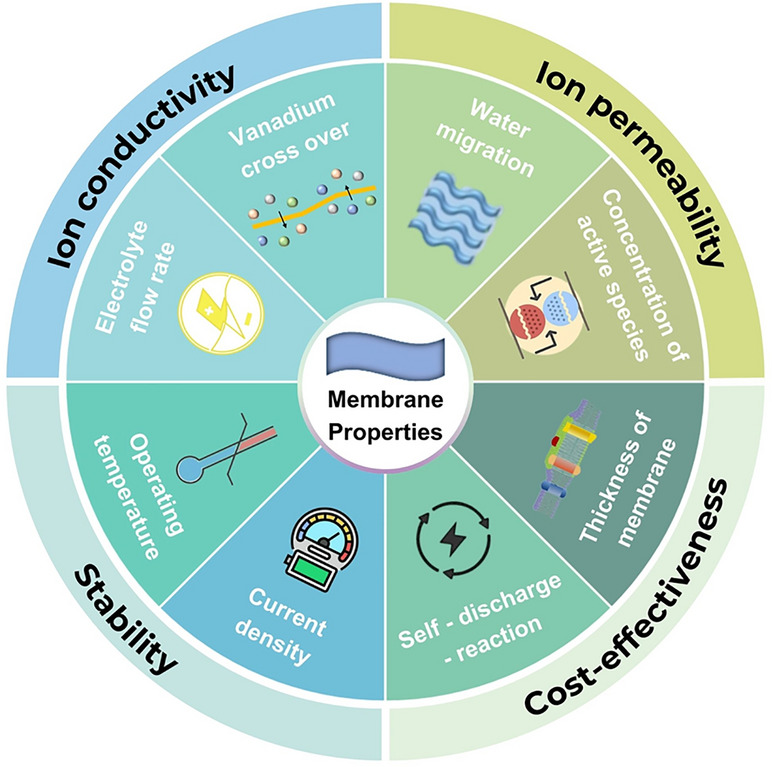

## Introduction

The increasing worldwide energy demand, environmental challenges stemming from the extensive consumption of fossil fuels, and the urgency to meet carbon emission targets are propelling the development of renewable energy technologies utiliing clean energy sources, such as solar and wind [1–6]. However, due to their inherent unpredictability and intermittency [7, 8], the electricity generated from renewable sources is difficult to be utilised directly and efficiently in practice. Large-scale energy storage systems are the key to facilitate the implementation of renewable energies by storing and then releasing a reliable energy supply when needed. Among the various storage technologies, redox flow batteries (RFBs) are anticipated to become a viable candidate for large-scale and long-duration applications. Initially conceptualised in the early 1980s, RFBs emerged as an innovative alternative to conventional battery systems due to their ability to decouple energy storage and power generation [9]. Unlike traditional batteries, where the electrochemical reactions take place within a single cell, RFBs rely on liquid electrolytes that are stored externally and circulated through electrochemical cells, allowing for easy scaling of both capacity and power [10]. The development of RFBs has been a significant advancement in the field of energy storage technologies. This expectation is based on their distinct features, including the ability of delivering both high energy capacity and high-power output, adaptable design, minimal toxicity, and extended cycle life [11–13]. To date, a variety of RFB systems have been developed, such as all-vanadium RFBs [14–16], zinc-based RFBs [17–19], iron-based RFBs [20], sodium-based RFBs [21], polysulfide-based RFBs [22], nitrobenzene-iodine RFBs [23], and aqueous organic RFBs [24]. Among them, the vanadium redox flow battery (VRFB) represents the most commercially viable RFBs.

VRFB was first proposed by Skyllas-Kazacos and colleagues in 1984 [25]. While the research and initial development of VRFBs began in the 1980s-1990s, their progress and commercial viability rose dramatically in the 2010s and especially recently, driven by the growing need for long-duration energy storage to support renewable energy integration and grid stability. Possessing all the typical properties of RFBs, VRFB also has the advantage of being environmentally friendly and economically efficient, making it suitable for large-scale energy storage applications. Unlike other RFBs, such as Zn-based or Fe-based batteries, VRFBs do not suffer from cross-contamination issues since they use vanadium ions on both positive and negative sides [26]. A VRFB consists of three critical components: electrodes, electrolytes, and a membrane. Numerous factors can affect the performance of VRFBs, including reactivity of the electrode [27–29], concentration and flow rate of the electrolyte [30, 31], operating temperature [32], stability of components, ionic conductivity, and vanadium ion permeability of the membrane [33]. As one of the key components in VRFBs, a membrane separates the active species of the positive and negative electrolyte while allowing charge carriers to pass through [34, 35]. The membrane directly affects the efficiency and stability of the battery. Three critical parameters of membranes determine the performance of VRFBs, including vanadium ion permeability, proton conductivity, and stability. Additionally, the cost-effectiveness of a membrane can significantly facilitate the commercialisation of VRFBs in the market. The proton conductivity of a membrane is primarily determined by the rate of proton transport, whereas ion permeability refers to the membrane’s capacity to block vanadium penetration [36]. Therefore, obtaining a deeper comprehension of the ion transport mechanism across the membrane is key to investigating suitable strategies to achieve a high-performance battery system.

In the past decades, the most extensively studied ion exchange membranes for VRFBs were based on perfluorinated sulfonic acid (PFSA) membranes. However, despite their high ion conductivity, the high ion permeability and cost of the commercial perfluorinated membranes posed significant challenges for the further development of VRFBs. Consequently, there is an urgent need to develop novel membranes with strong ionic conductivity, low vanadium ion permeability, high ion selectivity, exceptional chemical and mechanical strength, and cost competitiveness [37, 38]. Recent studies have highlighted the potential of advanced materials and fabrication technologies to enhance the overall performance as well as reduce both the cost and environmental impact of VRFBs. Therefore, the ongoing development of membrane technology is driven by the persistent innovation of novel materials, optimised structures, and enhanced functionalities that aim to address key challenges such as cost, efficiency, durability, and sustainability. The research is highly dynamic, and innovations in membrane design are expected to play a critical role in improving the performance, cost-effectiveness, and market viability of VRFBs. In this review, we will evaluate the ion transport mechanisms within membranes, identify the key parameters required for high-performance membranes, and discuss the current challenges of membranes for VRFBs. We also analyse and compare the ion conductivity, permeability and stability of different membranes. By evaluating the advantages and limitations of various materials and modification strategies for limiting ionic crossover and enhancing the performance of VRFB, we will present perspectives on the future development of next-generation membranes for VRFBs.

## Membranes for VRFB

### Working Principle of Membranes

Membranes utilised in RFB can be categorised into two types in general: i) ion exchange membranes (IEMs) and ii) porous membranes [34, 39]. According to the different functional groups fixed on the membrane, ion exchange membranes can be further classified into three types, including cation exchange membrane (CEM), anion exchange membrane (AEM), and amphoteric ion exchange membrane (AIEM). In addition, ion-solvating membrane (ISM) is a typical type of IEMs with a different ion transport mechanism, which is reviewed later in this article.

**Fig. 1:**
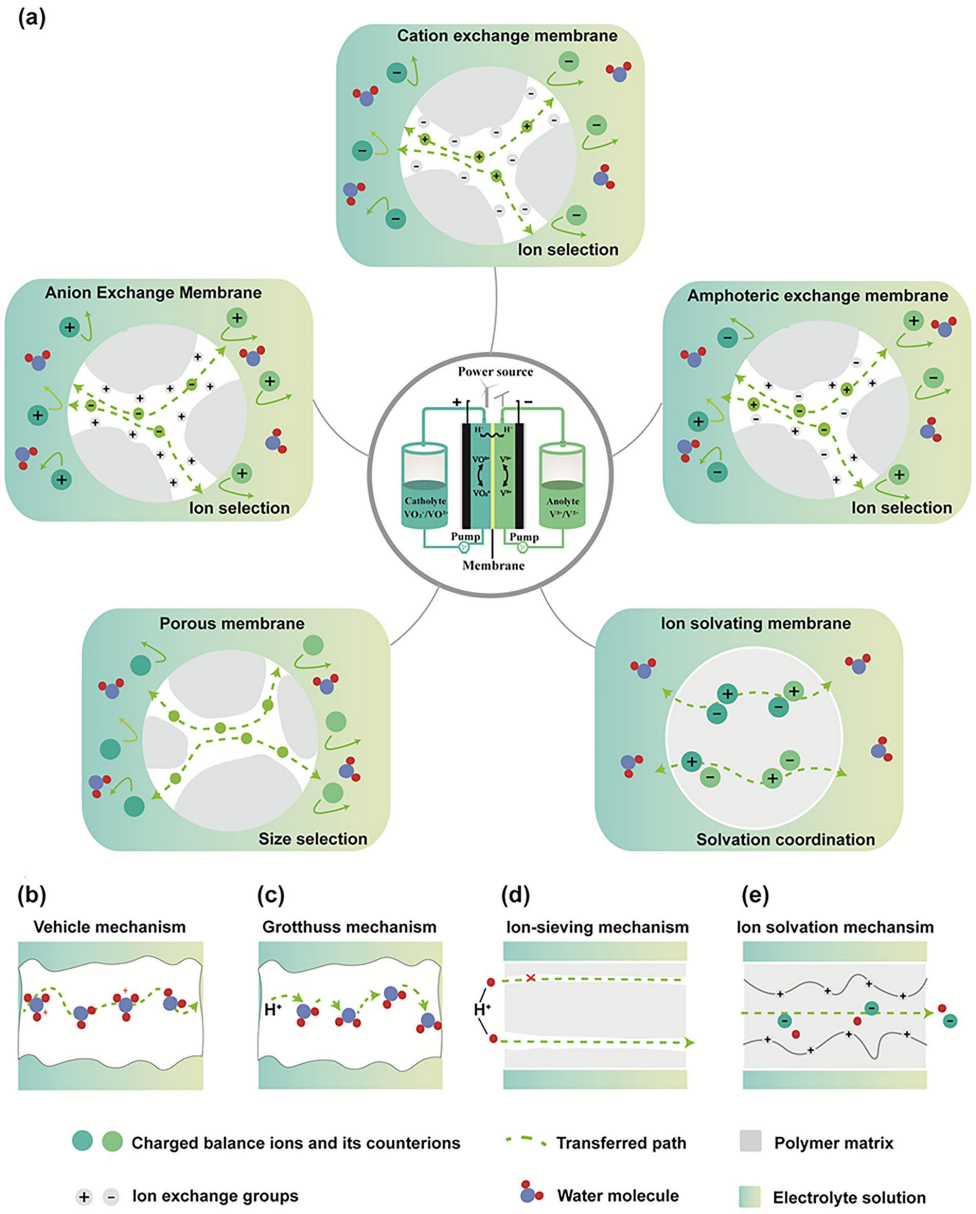
**a** Schematic demonstration of the VRFB and various membranes. Schematic illustration displaying **b** vehicle mechanism, **c** Grotthuss mechanism, **d** ion-sieving mechanism, **e** ion solvation mechanism

CEMs are composed of polymers with negatively charged functional groups, while AEMs are made of polymers with positively charged functional groups [36]. Due to their unique structures, each type of membrane can selectively exchange cations (*i.e.* H^+^, Na^+^, and K^+^) for CEM or anions (*i.e.* OH^−^, Cl^−^, and Br^−^) for AEM across the membrane. In IEMs, besides the exchange method, the charge carriers of electrolyte species can be transported through the membrane via the vehicle mechanism and Grotthuss mechanism [40]. In the vehicle mechanism, protons are mainly transported as hydrated hydrogen ions (Fig. 1b) while the Grotthuss mechanism, also referred to as the “hopping mechanism” involves protons transferring from hydronium donor sites to neighbouring acceptors (Fig. 1c) [41]. In membranes, these two mechanisms of proton transport can occur simultaneously. On the other hand, porous membranes are designed based on the principle of size exclusion [42]. In these porous membranes, micropores select ions through physical barriers. For example, in VRFBs, the radius of active species (VO_2_^+^/ VO^2+^, V^3+^/V^2+^) is greater than 0.6 nm [43], while the radius of charge carriers (H^+^, H_3_O^+^, SO_4_^2−^, and HSO_4_^−^) is less than 0.3 nm [44]. The difference in pore sizes enables the development of porous membranes by the ion-sieving mechanism (Fig. 1d). In contrast to the IEMs, which deliver ions based on the structure of microphase separation between hydrophilic matrixes with ion exchange groups and hydrophobic clusters [45], the ion transport channel in porous membranes is formed by the interconnected porous structure. Meanwhile, the ion conductivity of ISMs is achieved by the interaction between the polymer and electrolyte to form a homogeneous system [46]. In ISMs, the proton solvation is essential for proton transport. These membranes often contain water or polar groups that can donate or accept protons, hence facilitating the proton transfer process (Fig. 1e) [47].

In VRFBs, electrolyte imbalance during battery operation is the most critical issue leading to device failure. The transporting mechanism of vanadium ion crossover through the membranes includes diffusion, convection, and migration, in which convection predominates [48]. The ion transport via diffusion is due to concentration gradients, while migration caused by the electric field, and convection caused by pressure gradients. All these factors arise simultaneously during the charge and discharge processes that lead to the mixing of active species on both cell sides and various side reactions could occur. Consequently, a self-discharge process will happen, resulting in efficiency losses and irreversible loss of capacity, causing the failure of the battery cell. To minimise the effects of convection on the vanadium crossover rate, the pore size should be reduced to below 15 nm, while a further reduction in pore size (close to vanadium ion sizes) is required to reduce the effect of migration and diffusion on the vanadium crossover rate [48].

According to Sun et al. [49–51], the reactions involved in the electrolytes are described as follows:

In positive electrode:

1 $${\text{VO}}_{2}^{ + } + {\text{V}}^{3 + } \to 2{\text{VO}}^{2 + }$$

2 $$2{\text{VO}}_{2}^{ + } + {\text{V}}^{2 + } + 2{\text{H}}^{ + } \to 3{\text{VO}}^{2 + } + {\text{H}}_{2} {\text{O}}$$

3 $${\text{VO}}^{2 + } + {\text{V}}^{2 + } + 2{\text{H}}^{ + } \to 2{\text{V}}^{3 + } + {\text{H}}_{2} {\text{O}}$$

In negative electrode:

4 $${\text{V}}^{2 + } + {\text{VO}}^{2 + } + 2{\text{H}}^{ + } \to 2{\text{V}}^{3 + } + {\text{H}}_{2} {\text{O}}$$

5 $${\text{V}}^{2 + } + 2{\text{VO}}_{2}^{ + } + 2{\text{H}}^{ + } \to 3{\text{V}}^{3 + } + {\text{H}}_{2} {\text{O}}$$

6 $${\text{V}}^{3 + } + {\text{VO}}_{2}^{ + } \to 2{\text{VO}}^{2 + }$$

Generally, the crossover is asymmetric, thus leading to an imbalance of the electrolyte between the two sides of the cell. The diffusion coefficients of vanadium ions across the CEM were reported to follow the order: V^2+^  > VO^2+^  > VO_2_^+^  > V^3+^ [49]. The amount of V^2+^ and V^3+^ ions transferred has been calculated to be larger than that of VO_2_^+^ and VO^2+^ ions, resulting in the net vanadium crossover tending to move in the direction from anolyte to catholyte, the vanadium ions concentration imbalance and causing capacity loss with cycling. Meanwhile, the AEMs have been reported to reduce vanadium ion crossover due to the lower diffusion coefficients of vanadium ions in the membranes compared to CEM. The diffusion coefficients of vanadium ions across AEM have been found in the order: V^2+^  > VO_2_^+^  > V^3+^  > VO^2+^ [51]. The reduced vanadium ion permeability of AEM can be attributed to the Donnan exclusion phenomenon, which lessens the transfer of positively charged active species [52].

**Fig. 2:**
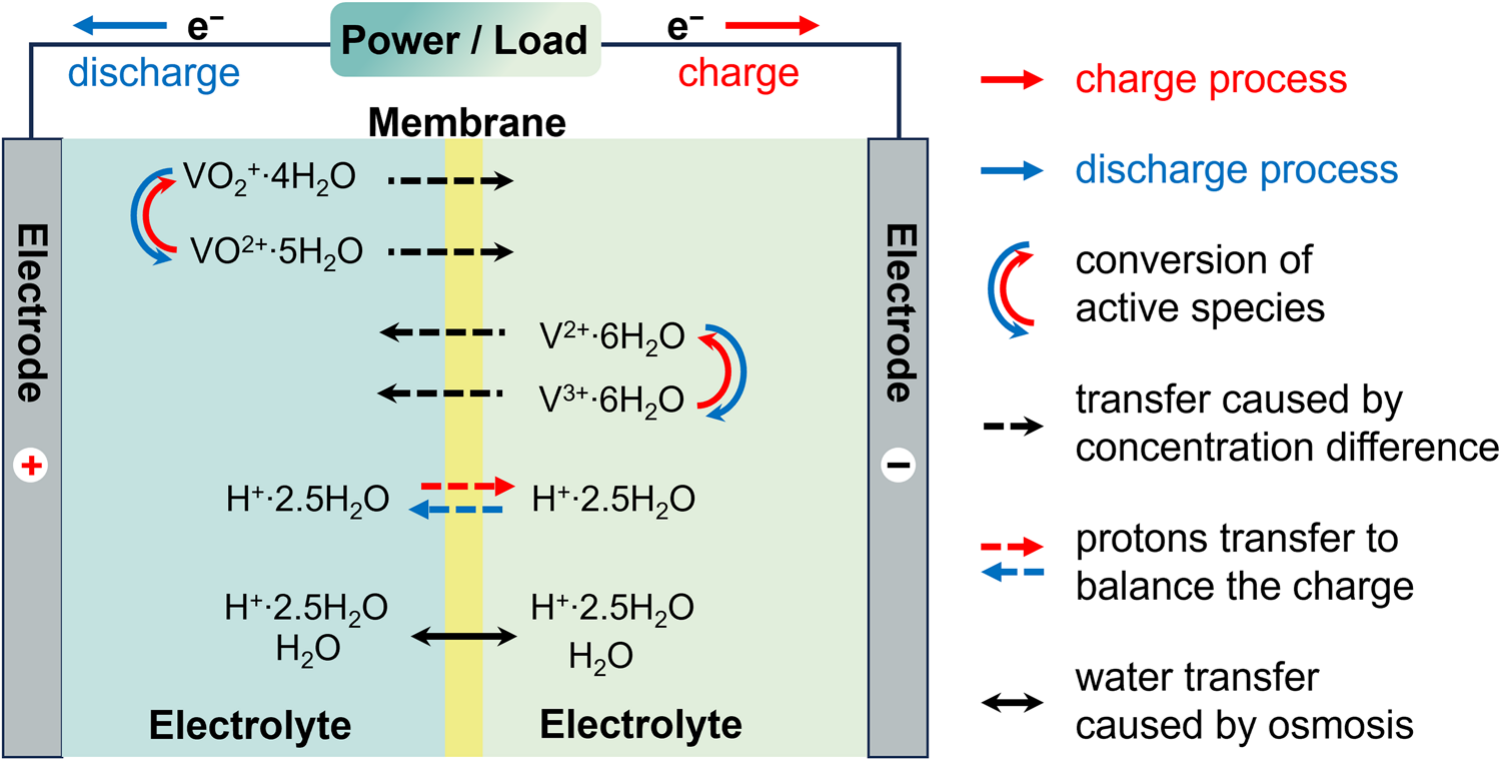
Illustration of mass transport across the membrane during the charge–discharge process

In addition to the migration of the active ions, water transport also strongly influences the volumetric changes of the electrolytes. During the charge process (see Fig. 2), oxidation occurs at the positive electrode, and electrons move from the positive electrode to the negative electrode through the external circuit. As electrons move, an equivalent number of protons must follow along with water molecules (*i.e.* hydrated form), moving to the negative side to maintain electrical neutrality inside the internal circuit. In contrast, during a discharge process, the proton moves with the dragged water from the anolyte to the catholyte, resulting in water transfer towards the catholyte. Furthermore, the transport of active species across the membrane induces the migration of bound water within the hydrated shells. In VRFB, both V^2+^ and V^3+^ ions are surrounded by six water molecules [53], while VO^2+^ and VO_2_^+^ have five and four water molecules in their hydrated shells, respectively [54]. It is important to note that the movement of other electroactive species in supporting electrolytes also facilitates the transport of protons to maintain charge neutrality, hence promoting the movement of dragged water. Lastly, the water molecules can be migrated by osmosis as a result of the existence of the electric field [11] that contributes significantly to the changing of water transport direction during the charge and discharge process [55]. Under the combined effect of the above processes, the water volume on both sides of the battery’s electrolytes becomes unbalanced.

The most common approach for minimising the resulting capacity fading of VRFBs due to the imbalance of the electrolyte is by improving the ion selectivity of the membranes. The IEM performs an essential role in the VRFB, acting as a proton conductor as well as a stable barrier for vanadium ions permeation. If the membrane is damaged (or possesses substantial pores), it will allow active ions on both sides to move freely across the membrane and can even allow direct contact between the positive and negative electrodes, leading to a short circuit. Consequently, the cell suffers from a drop in overall efficiency, and sudden capacity fades over cycling and failure. Conversely, if the membrane is too dense, it will restrict the transport of balancing ions, resulting in a reduction in the cell’s capacity.

### Properties of an Ideal Membrane for VRFB

Since the membrane’s properties determine the crossover behaviour, developing a membrane with superior properties that can inhibit the vanadium ion crossover is critical for VRFB. To achieve a high-performance VRFB, an ideal membrane should possess the following properties:

1. High ion conductivity. High power density is an important performance parameter for VRFBs that requires a high ion conductivity (in VRFB it is usually the proton conductivity) of the membrane. To achieve this, reducing internal resistance caused by the membrane is extremely important to prevent Ohmic polarisation and energy loss. Hence, membranes with sufficient ion transport channel structure to promote ion conduction are essential, allowing fast ion transportation to complete the redox couple reaction [56]. In addition, the proton conductivity also affects the capacity and efficiency (*e.g.* voltage efficiency (VE) and energy efficiency (EE)) of VRFB, especially at high current densities, potentially resulting in considerable capacity loss and a significant drop in efficiency. The conductivity of the membrane in a VRFB is governed by several factors, including the polymer matrix, ion exchange capacity, and hydration level.
2. High ion selectivity. Generally, the ion selectivity is defined as the ratio of the value of ionic conductivity to the value of vanadium ion permeability. Ion selectivity is a crucial characteristic of membranes, allowing certain ions to pass through while blocking others. The permeability of a membrane depends on its inherent structure and properties, including the size of ion channels, the density of ion exchange groups and their charge type, as well as the hydrophilicity of the membrane matrix [57]. High ion selectivity is particularly important for VRFB systems as it reduces the side reactions in the electrolyte to reduce self-discharge and capacity loss. It is worth noting that the high proton conductivity will inevitably deliver high performance of VRFB (high Coulombic efficiency (CE) and EE) by enhancing ion selectivity. By definition, any factor that can affect the ion conductivity and vanadium ion permeability of a membrane can influence ion selectivity. The ion selectivity is mainly influenced by the microstructure of the membrane, including the polymer matrix, the pore size, and the ion exchange groups, the ion exchange capacity, and the charging state of membrane. A membrane with a precise pore size may differentiate ions according to their dimensions, facilitating selective ion transport. In addition, the presence of functional groups on the membrane influences its ability to interact with specific ions which affect the ion selectivity of the membrane.
3. Superior mechanical properties. As the membrane in VRFBs will endure pressure during the assembly and battery operation, it must possess strong mechanical strength. According to recent studies, mechanical stresses cause more detrimental effects on the membrane than chemical degradation [58]. The mechanical characteristics of a membrane can be determined by two parameters: tensile strength and swelling ratio. Tensile strength is evaluated by analysing the stress–strain test while the swelling ratio is measured by comparing the dry and wet membranes [59]. Superior mechanical properties help membranes resist various stresses during its life span, ensuring good cycling stability of the VRFBs. If the membrane is damaged by stress, it will allow the positive and negative electrolytes to flow freely, causing side reactions and rapid decay of capacity and efficiency, ultimately leading to battery failure. In VRFB, the membrane swelling rate is mainly examined by the extent of membrane expansion by water (aqueous solution) absorption. High water absorption facilitates proton conduction via membranes. However, the high swelling rate of the membrane also promotes the vanadium ions penetration, reducing the stability of membranes. The type of polymer or material used in the membrane (*e.g.* PFSA, sulfonated hydrocarbon polymers, porous membrane) significantly affects its mechanical properties. Controlling the modification strategies (degree of cross-linking, degree of sulfonation, porosity) can effectively enhance the mechanical stability of the membrane.
4. Excellent chemical stability. Due to the strong acidic (e.g. H_2_SO_4_ and HCl) and oxidising (VO_2_^+^) environment inside the VRFB, the membrane must possess excellent chemical stability to ensure its expected life span of the VRFB (10–20 years). This harsh environment degrades the structure of the polymer and destroys the membrane, resulting in a deterioration of the capacity and efficiency of the VRFBs, thereby reducing its cycle stability. Similar to physical stability, the chemical stability of the membrane is influenced by the material selection and the modification techniques. Based on the strong acid and oxidising environment during VRFB operation, if the membrane has better protection against oxidation and corrosion, its chemical stability will be stronger. For example, the PFSA membranes with excellent chemical stability is due to the fact that their polymer backbone is well protected by fluorine groups, thus avoiding damage by free radical attack.
5. Cost-effectiveness. The cost of Nafion membranes, the widely used membranes for VRFB, is as high as 500–1000 USD per m^2^ [60], which considerably increases the cost of VRFBs, limiting the broad application of VRFB systems. In addition, the commercialised perfluorinated membranes also suffer from high vanadium ion crossover [61]. Hence, it is imperative to develop more cost-effective alternative membranes for VRFB. The type of material used in the membrane and the complexity of the membrane fabrication process have a significant impact on cost. Advanced materials and techniques can increase production costs due to the need for specialised equipment, chemicals, and labour. Furthermore, its thickness is also an important factor, impacting both cost and performance of the membrane.

### Challenges of Membranes for VRFBs

Theoretically, the performance of VRFB systems can be enhanced by improving conductivity of protons and simultaneously minimising the permeability of vanadium ions. The difficulty in enhancing the selectivity of an IEM stems from the fact that conductivity and permeability are somewhat correlated since both parameters depend on the movement of ions through the membrane. Modifications of IEMs aimed to improving their conductivity are often accompanied with increased vanadium ion permeation. Conversely, lowering the active ions crossover invariably leads to a decrease in proton conductivity. As a result, most efforts to enhance the ion selectivity of membranes focus on either increasing proton conductivity or decreasing the active ions crossover while striving to maintain the other parameter. There are two main ways to enhance the performance of membranes via modifying PFSA membranes or synthesising non-fluorinated membranes [35]. These approaches aim to address the critical challenges associated with achieving an ideal balance between ionic conductivity and permeability, while enhancing the chemical durability of the membrane. By improving those fundamental properties, the goal is to develop membranes that not only exhibit high ion transport efficiency but also maintain long-term performance and ensure cost-effectiveness for large-scale VRFBs.

Commercial PFSA membranes are commonly utilised in VRFBs because of their high ion conductivity and excellent chemical stability. But they suffer from low ion selectivity due to the high vanadium ion permeability [49, 62]. Many modification methods have been reported to enhance ion selective of the Nafion membranes, such as addition of additives like SiO_2_ [63], TiO_2_ [64], graphene oxide (GO) [65], and lignin [49, 62, 66]. Furthermore, the Nafion membrane can be blended with other polymers such as sulfonated poly (ether ether ketone) (SPEEK) [67], polytetrafluoroethylene (PTFE) [68], polyvinylidene difluoride (PVDF) [69], and polypropylene (PP) [70]. Although these modified membranes successfully decrease the permeability of the vanadium ions, the difference in hydrophilicity and swelling ratio among the various components may cause microphase separation in the membranes. Since most of the additives are hydrophobic, causing discontinuous or poorly connected hydrophilic domains inside the membranes, which in turn reduces the continuity of ion transport channels and may also increase membrane resistance [69, 71]. In addition, the low swelling ratio caused by hydrophobic components may narrow the size of the water channel. Both circumstances substantially increase the ion transport barrier, resulting in decreased proton conductivity. Additionally, blending another material into the polymer matrix increases the thickness of membranes, a critical property of the membrane, which influences both cell function and membrane cost [62]. A thicker membrane will have lower permeability and higher mechanical strength; however, it also increases the area resistance as well as the cost of the membrane. Lastly, excessive additives (*e.g.* carbon-based materials) may lead to a short circuit between the cathode and anode, potentially causing cell failure.

To mitigate the high cost and decrease the vanadium ion permeability of the PFSA membranes, several alternative membranes have been developed using hydrocarbon polymers with more cost-effective and lower permeability, including sulfonated poly(ether ether ketone) (SPEEK) [72, 73], sulfonated poly (arylene ether) (SPAE) [74], sulfonated poly(arylene ether ketone) (SPAEK) [75], sulfonated poly(phthalazinone ether ketone) (SPPEK) [76], and sulfonated polyimide (SPI) [77]. These hydrocarbon polymers possess rigid structures with benzene rings, which benefit from π-π bonding, providing excellent chemical stability. Moreover, hydrophilic groups can improve the wettability of the membrane to promote proton conductivity [34]. Many strategies have been reported to enhance hydrocarbon membranes performance, such as blending, grafting, cross-linking, and porous substrate filling. Introducing functional groups into a polymer matrix creates additional pathways for ion transportation, including proton conduction. However, the radicals could attack the polymer matrix, causing degradation of the membrane. It indicates that introducing ion exchange groups into the polymer matrix reduces the long-term stability of the polymer. Hence, new modification methods are needed to balance the performance and stability of the membranes. In the following, we will review the advancement of VRFB membranes through a typical literature analysis based on the classification of IEMs, AIEMs, porous membranes, and ISMs.

## Advanced Membranes for VRFBs

### Modification Materials and Methods

**Fig. 3:**
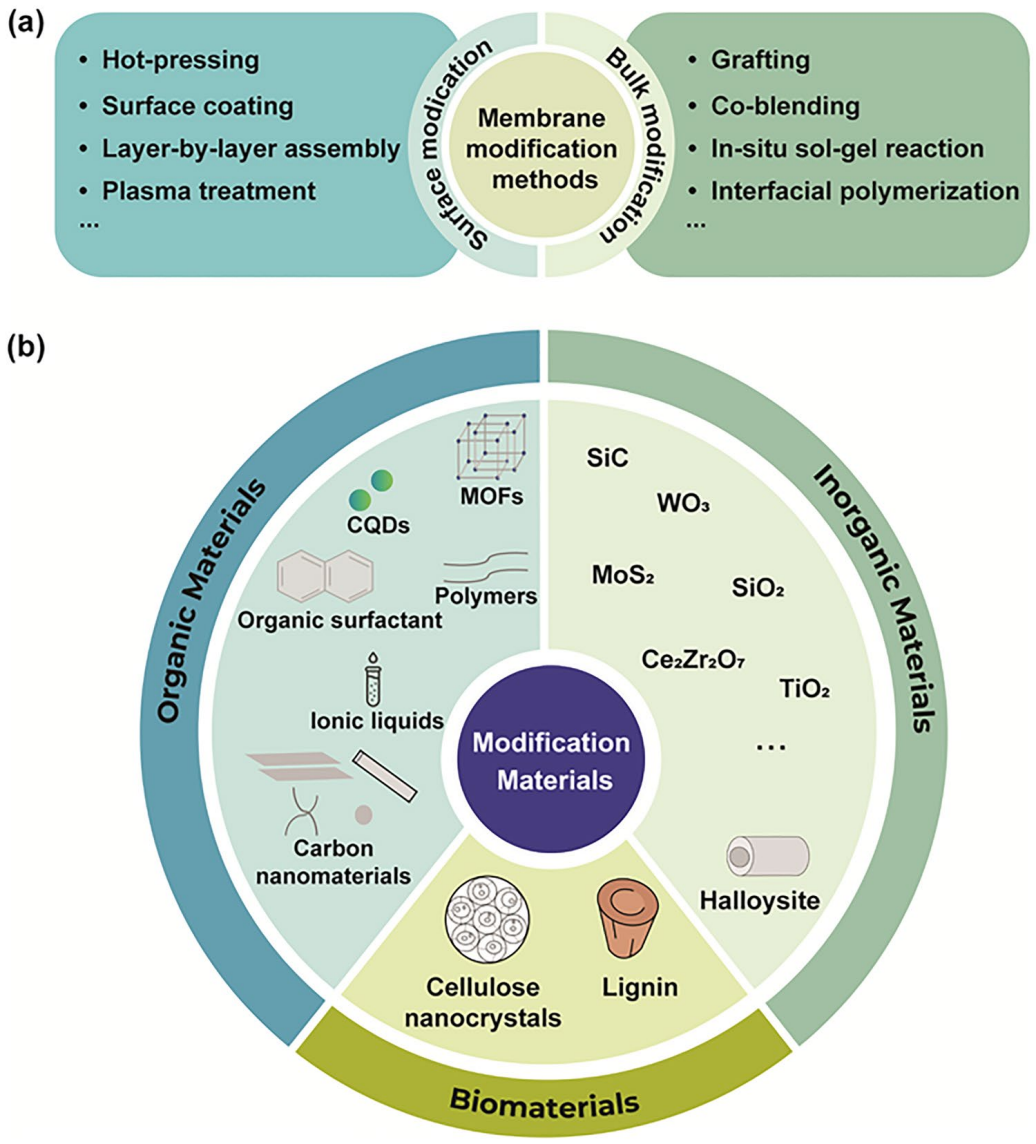
**a** Summary of membrane modification methods. **b** Material selection for membrane modification

As mentioned above, vanadium ion permeability is a critical issue causing the irreversible capacity fading of VRFBs. Therefore, it is necessary to optimise the membranes to decrease the vanadium ion permeability, increase the ion selectivity, and promote the VRFB performance. To date, various innovative methods have been exploited to improve the membrane properties in VRFBs. Composite membranes contain two or more components, which are commonly organic materials, inorganic nanomaterials, or multiple composite polymers. Figure 3a summarises a variety of materials used for the fabrication of composite membranes. Meanwhile, the modification method can be classified into two main types: surface modification and bulk modification (Fig. 3b). Surface modification involves modifying the surface properties of a membrane to improve its performance via methods such as coating, chemical grafting, plasma treatment, layer-by-layer assembly, and hot-pressing. This process can enhance characteristics such as hydrophilicity, ion selectivity, and thermal and chemical stability. Unlike surface modification, which only affects the outer layer of the membrane, bulk modification changes the internal structure and composition of the membranes to achieve unique properties. The common methods used in the bulk modification include co-blending, cross-linking, solgel reaction, and polymerisation techniques such as the phase inversion method or interfacial polymerisation. Based on these approaches, numerous composite membranes have been fabricated with improved performance.

### Ion Exchange Membranes

**Fig. 4:**
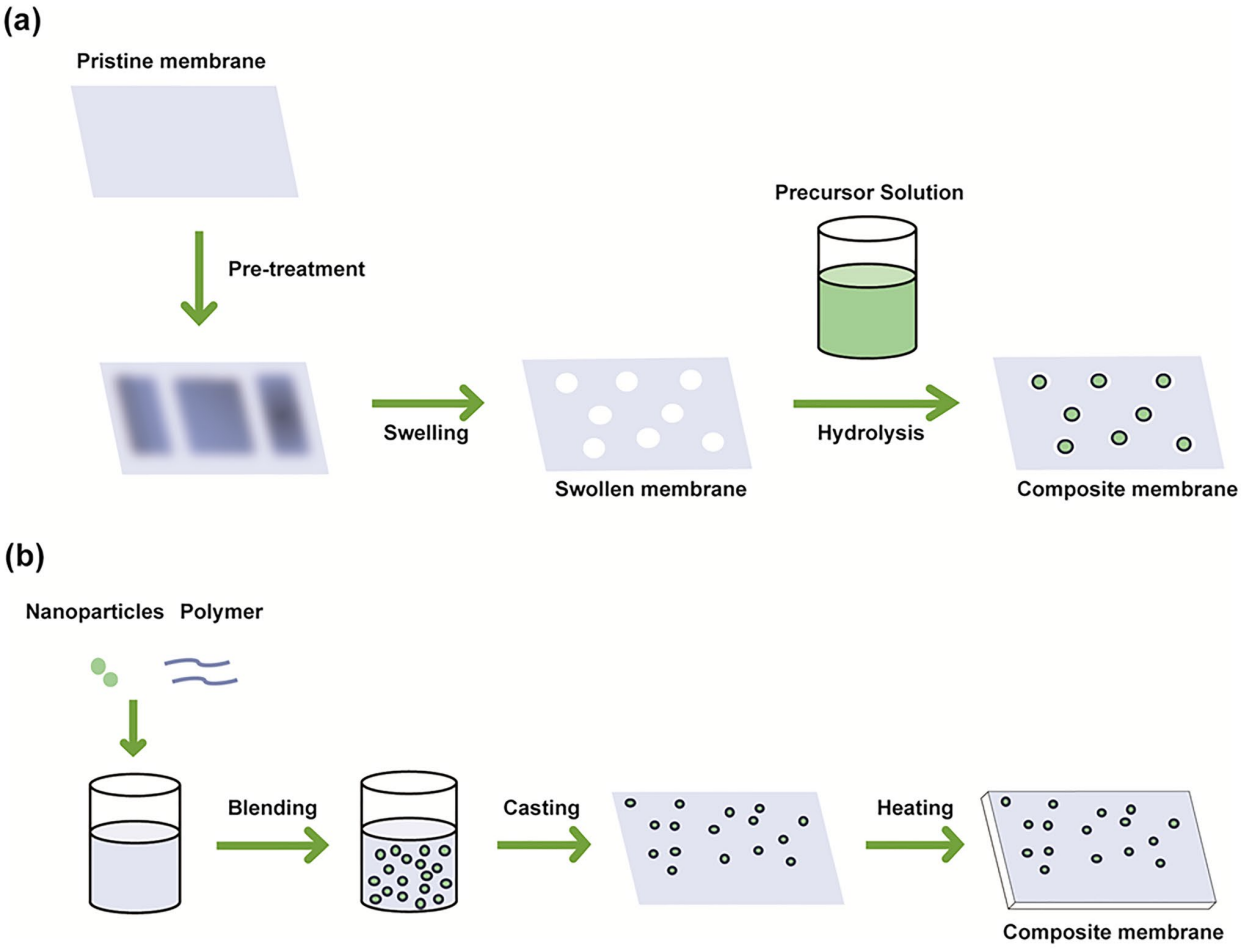
Schematic process of the composite membrane synthesis by: **a** in situ solgel method, **b** physical blending method

The ion-conductive mechanism of IEMs relies on the transit of ions via the membrane’s ion-conducting channels, enabled by its fixed charged groups. This mechanism integrates electronic interactions, hydration, and ion diffusion to facilitate ion transport. To improve the performance of IEMs, enhancing the ion conductivity while reducing vanadium ion permeability is a crucial requirement. One of the most effective approaches is by mixing the polymer matrix with other polymers or organic/inorganic nanomaterials to create composite membranes. These composite membranes offer superior performance compared with the original membranes, such as reduced migration rate of vanadium ions and improved mechanical strength. Generally, the nanofillers are embedded into the polymer matrix via two techniques: the in situ solgel method and the co-blending method. The solgel process (Fig. 4a) is a method for producing solid materials from small molecules. The solgel method involves two main reactions: (1) the hydrolysis of the precursor in the acidic or basic mediums and (2) the polycondensation of the hydrolysed products [78]. As a consequence, a polymeric network is formed in which nanofillers can be retained [79]. The main advantage of this method lies in that it allows the chemical formation and incorporation of nanofillers within the polymer matrix, leading to better particle dispersion and stronger interactions. Comparatively, the co-blending method is considered a simpler method, in which the nanofillers are physically blended and dispersed into the polymer dispersion, which is then processed to form a composite membrane. In contrast to the solgel method, the co-blending method (Fig. 4b) involves the physical mixing of pre-formed particles with the polymer, which may result in less uniformity and weaker interfacial interaction [80]. The recent advancements in IEMs used for VRFBs are reviewed in the light of the types of additives used to modify the membranes including inorganic, carbon-based, organic, polymers, and biomaterials.

#### Inorganic Materials

**Fig. 5:**
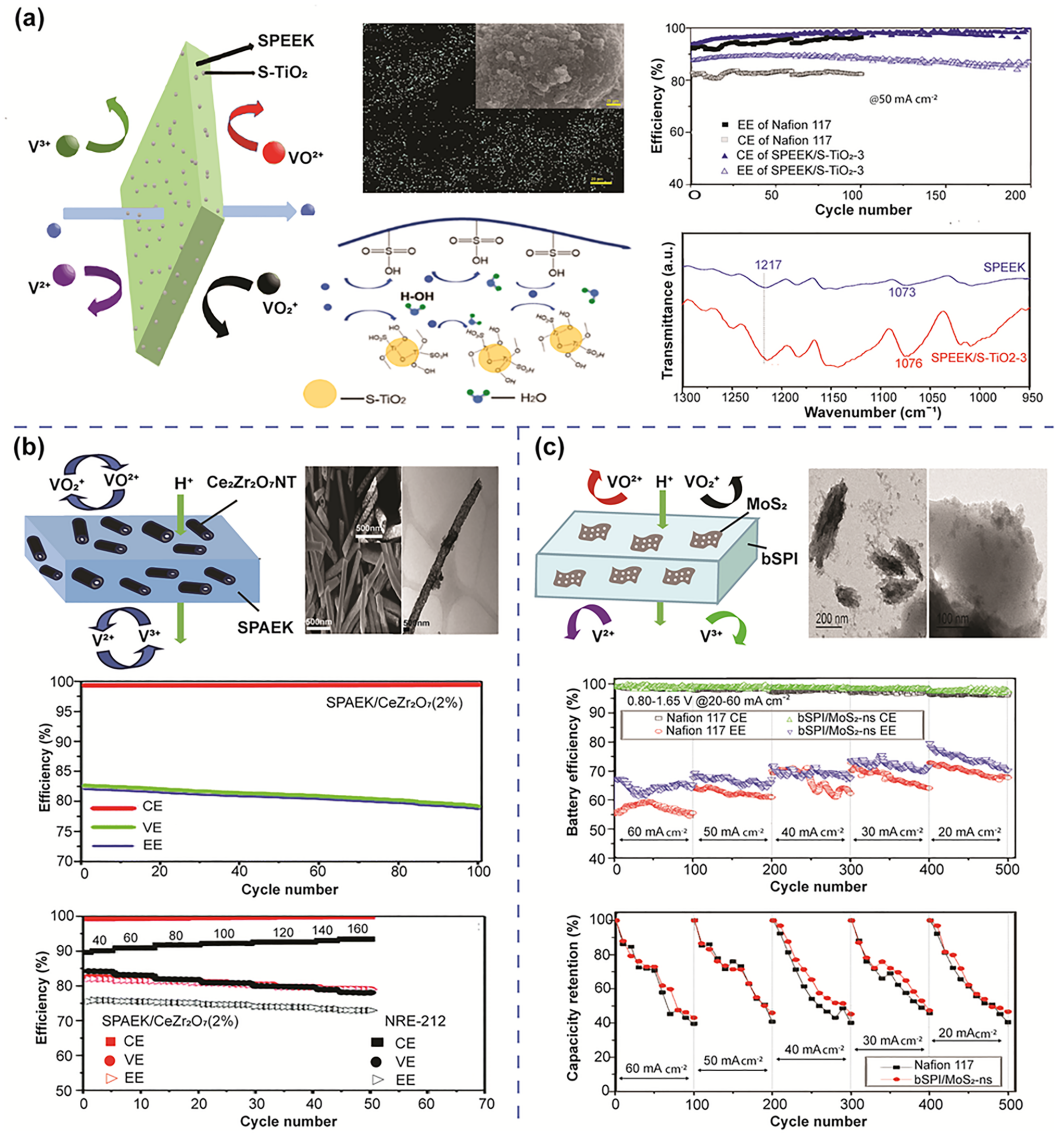
**a** Schematic illustration of composite membranes with inorganic materials: The composite membrane with nanoparticles, EDX element mapping images of S-TiO_2_ nanoparticles; battery performance of VRFB cell using the composite membrane, reproduced from Ref. [83] with permission from Elsevier, copyright 2022. **b** Composite membrane with Ce_2_Zr_2_O_7_ nanotubes, TEM image of Ce_2_Zr_2_O_7_ nanotubes, battery performance of VRFB cell using the composite membrane, reproduced from Ref. [84] with permission from Royal Society of Chemistry (RSC), copyright 2018. **c** Composite membrane with MoS_2_ nanosheets; TEM image of exfoliated MoS_2_ nanosheets, battery performance of VRFB cell using the composite membrane, reproduced from Ref. [87] with permission from Elsevier, copyright 2018

Inorganic materials (*e.g.* nanoparticles of non-metal oxide or transition metal oxide) are considered a promising class of materials for enhancing the ion selectivity of IEMs. Over the past decades, there were many types of membranes containing inorganic nanofillers were successfully fabricated. In 2007, Xi et al. [63] incorporated silica nanoparticles uniformly into a Nafion 117 membrane via an in situ solgel method. Consequently, the vanadium ion permeability of the modified membrane was markedly decreased compared to the pristine Nafion 117 membrane. A negligible reduction in the proton conductivity of the composite membrane is attributed to the compatibility of the hydrophobic SiO_2_ particles with the polymer backbone of the Nafion 117 membrane. The silica nanofillers were also successfully incorporated into an AEM (Fumasep FAP) to address the trade-off of vanadium ion crossover and proton conductivity [81]. The vanadium ion permeability of the modified membrane decreased by 20% and 25% compared to the pure AEM and the Nafion membrane, respectively. Consequently, compared to the batteries employing pristine AEM and Nafion, the AEM with silica nanocomposite produced an 88% CE at 20 mA cm^−2^, which was 3% and 7% higher, respectively. Nevertheless, a higher area resistance was still observed after silica modification due to the lack of charge carrier availability in silica nanofillers, disrupting proton transport pathways. To solve this issue, the nanofillers could be functionalized to further improve the membrane proton conductivity. In 2019, Wang et al. [82] prepared a composite membrane by blending sulfonated titanium dioxide (s-TiO_2_) nanoparticles into the SPEEK matrix (Fig. 5a). Adding −SO_3_H groups to TiO_2_ nanoparticles was found to provide Bronsted acid sites in the polymer matrix, which interacted with –OH groups on the neighbouring Ti^4+^ ions, forming stable coordination bond and considerably enhancing the interfacial compatibility with the membrane matrix. The existence of numerous hydrophilic groups, including –OH and –SO_3_H coexisting in the s-TiO_2_ nanofillers, greatly enhanced their water uptake and IEC. This was attributed to the increase in proton carriers by increasing the –SO_3_H groups in the membrane matrix that facilitate proton transfer. Meanwhile, the membrane incorporated with s-TiO_2_ nanofillers also increased the barrier to reject vanadium ion permeation. The s-TiO_2_ nanofillers balanced vanadium ion permeability and proton conductivity thereby improving the ion selectivity of the membranes. Accordingly, the cell of s-TiO_2_’s membrane had an 82% EE at 50 mA cm^−2^, which is much higher than that of the pristine SPEEK membrane and SPEEK/TiO_2_ composite membrane. In 2022, Zhang et al. [83] investigated the effect on the physicochemical properties of the SPEEK/s-TiO_2_ membrane to determine the optimal concentration of s-TiO_2_ in the membrane, thereby enhancing the performance of the VRFB cells. It was shown that at 3 wt% loading of s-TiO_2_, the hybrid membrane demonstrated comparable permeability and selectivity compared to other ratios.

The 1D inorganic nanofillers (nanotubes) and 2D inorganic nanofillers (nanosheets) were also employed as additives to boost the membranes’ ion selectivity. Hossain et al. [84] developed a composite membrane by introducing cerium zirconium oxide nanotubes (Ce_2_Zr_2_O_7_NT) into sulfonated poly(arylene ether ketone) (SPAEK) polymers to make SPAEK/Ce_2_Zr_2_O_7_ composite membranes (Fig. 5b). Superior durability against oxidant agents and acid/base environment was obtained with the composite membranes, which contributed to the excellent water adsorption and good retention capacity. Compared to pure SPAEK and commercial Nafion212 membranes, the modified membrane demonstrated 12- and 27-times lower vanadium ion permeability respectively. Ye et al. [85] recently introduced super-hydrophilic TiO_2_ nanotubes into the Nafion membrane matrix, resulting in a hybrid membrane with outstanding performance and exceptional stability. The chemical interaction between the nanotubes and the polymer matrix, facilitated by the bonding of Ti^4+^ ions at the TiO_2_ surface to –OH groups in water, enhanced the hydrophilicity of the material, making it easier to disperse in the membrane matrix. Similar to TiO_2_ nanoparticles, TiO_2_ nanotubes possessed a strong affinity to the polymer matrix due to the binding between Ti^4+^ ions and –SO_3_H groups. Additionally, because the TiO_2_ nanotubes blocked and thus elongated the vanadium ion diffusion pathway, the Nafion/TiO_2_ nanotube hybrid membrane showed lower vanadium ion permeability. Furthermore, the attachment and orientation of –SO_3_H groups on the TiO_2_ nanotube surface promoted proton transportation, hence improving ion selectivity of the membrane.

2D materials consisting of a single or few layers of atoms have been commonly employed to modify IEMs for VRFB. Their unique properties, including high surface area and mechanical strength, make them valuable additives in composite membranes [86]. The unique composition and characteristics of nanosheets provided a stable barrier against vanadium ion crossover, enhancing both mechanical and chemical durability of the membrane. For example, by embedding molybdenum disulfide (MoS_2_) layer into branched sulfonated polyimide (bSPI), Pu et al. [87] reported a novel composite membrane (bSPI/MoS_2_-ns) that exhibited low vanadium ion permeability and good ion selectivity. The synergistic effect between hydrated − SO_3_H groups in the polymer and the hydrated mesoporous MoS_2_ nanosheets absorbing water molecules created more continuous proton transport routes, improving proton transportation. Furthermore, the addition of MoS_2_ nanosheets with a robust 2-dimensional layered structure minimised the vanadium ions permeation (Fig. 5c). As a result, the composite membrane also exhibited an outstanding performance with high CE (99%) and EE (80%) in a long lifespan (over 500 cycles). Silica carbide (SiC) is also a 2D semiconductor material with high mechanical stability, which has been used to modify non-fluorinated sulfonated polymer membranes. Incorporation of SiC may increase the mechanical and chemical stability of membranes. Zhang et al. [88] used sulfonation and amination techniques to functionalise SiC nanosheets to further enhance their properties in the membrane. The SiC nanosheets play two roles in the membranes. First, the served as physical barriers to prevent crossing of vanadium ions. Secondarily, the –NH_3_^+^ groups on the surface of SiC repelled vanadium ion permeation via the Donnan exclusion effect, hence reducing the vanadium crossover. Although the proton transportation was limited because of the block effect of the addition of SiC nanosheets, the protons were transported across the membrane via narrow channels created by the electrostatic interaction between –SO_3_H and –NH_2_. Consequently, the enhanced proton conductivity while reducing vanadium permeability yielded an excellent ion selectivity of 2.99 × 10^5^ S min cm^−3^, which greatly exceeded that of the Nafion 117 membrane (0.34 × 10^−5^ S min cm^−3^).

#### Carbon-Based Materials

**Fig. 6:**
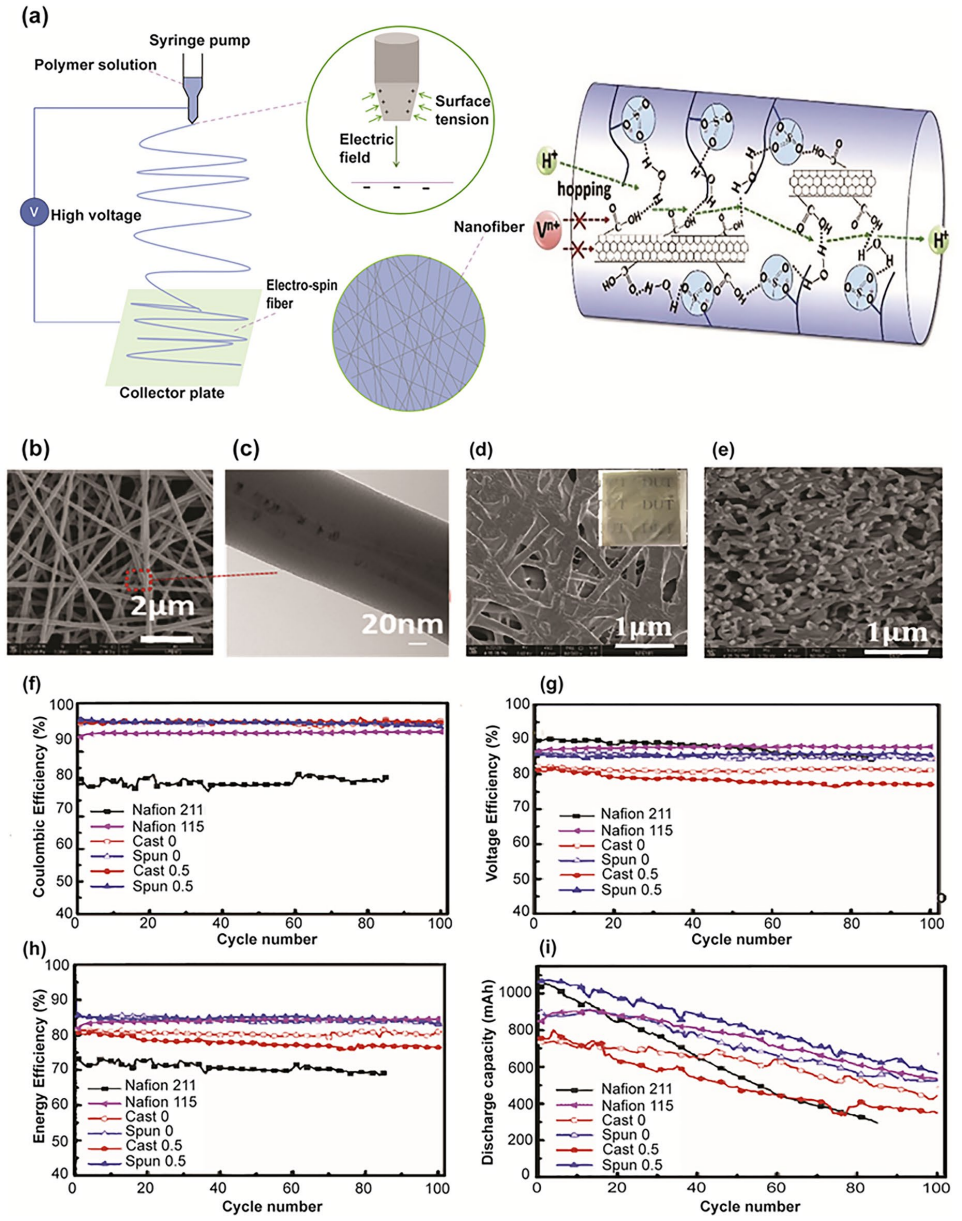
**a** Schematic process of membrane synthesis using electrospinning fiberisation method. **b** Morphologies of different nanofibers. **c** TEM image of the orientation of carbon nanotubes in the SPEEK electrospun nanofiber. SEM image of the **d** surface and **e** cross section of carbon nanotubes/SPEEK membrane. **f** CE, **g** VE, **h** EE, **i** discharge capacity decay of the battery cell, reproduced from Ref. [89] with permission from Elsevier, copyright 2019

Besides inorganic materials, carbon-based materials, *i.e.* graphene oxide (GO) nanosheets and carbon nanotubes (CNTs), were also used to promote the performance of IEMs. Carbon-based materials provided abundant and effective active sites for ion adsorption and transportation, thereby improving the overall ion conductivity of the membrane. Current research focuses on further modifying properties of carbon materials to improve their characteristics by grafting surface functioning carboxyl, hydroxyl, or other hydrophilic groups. Li et al. [89] introduced an innovative approach involving electrospinning fiberisation of the multi-walled carbon nanotubes to improve hydrophilic interconnection and vanadium ion blocking in the composite membranes, thereby achieving elevated proton conduction and reduced vanadium ion permeability (Fig. 6a). The high electrospinning potential was proven to have a great impact on the preparation of multi-walled carbon nanotubes and the fabrication of composite membranes. Under a strong electric field, the multi-walled carbon nanotubes coordinated within the nanofiber axis and showed excellent dispersion in the nanofibers with a diameter of around 20–30 nm (Fig. 6b, c). In addition, at high temperatures, the electrospun nanofibers gradually stuck to each other to form interconnecting networks (Fig. 6d, e). The results showed that the proton conductivity was enhanced with the excellent EE and VE (Fig. 6g, h) compared to Nafion membrane as the formation of interconnected proton pathways, facilitated by the electrospun nanofibers. In addition, the carboxyl group on the multi-walled carbon nanotubes can interact and scatter uniformly within the nanofibers under a strong electrospinning electrostatic field, facilitating the development of hydrogen bonding for proton transport. At the same time, the vanadium ions were inhibited from transition owing to the incorporation of the nanotubes, resulting in improved CE and long-term stability (Fig. 6f, i). In view of this, the electrospinning fiberisation technique offered a viable way to accomplish IEMs with high ion selectivity. Furthermore, Sigwadi et al. [90] reported that adding ZrO_2_-CNT in the Nafion 117 matrix improved the water uptake and swelling ratio of the membrane. The incorporation of CNT and ZrO_2_ increased the hydrophilicity domain on the surface of CNT which enhanced the membrane’s water uptake by 22% compared to the Nafion membrane. The modified membrane provided more active acid sites that promoted proton transportation through the membrane. The introduction of ZrO_2_ into the CNT also increased the barriers to prevent vanadium ions permeation, thus maintaining the ion selectivity of the membrane.

Graphene oxide (GO), a novel carbon-based material derived from oxidation of natural graphite or carbon nanofibers, presents a promising addition that could reduce permeability and improve the performance of IEM. GO has abundant oxygen functional groups on the surface of the fundamental planes and sheet edges, including hydroxyl, carboxyl acid, epoxy, and ketone [91] that can form interconnected networks within the polymer matrix, facilitating faster and more efficient ion transport. In addition, GO has a high surface area and a unique 2D layer structure, which allows protons to transport more freely, thereby improving the proton conductivity significantly. In 2014, Lee et al. [92] prepared a Nafion 117/GO hybrid membrane using the casting method. The generation of various oxygen functional groups expanded the inter-planar space of GO compared to that of pristine graphite, which increased the water retention capacity. However, in the modified membrane, the investigation indicated that the inter-planar spacing of the membrane was reduced, but the density of − SO_3_H groups inside the membrane rose, causing an increase in the IEC but a decrease in water-containing capacity. The reduced inter-planar planar spacing in the composite membrane also led to decreased water absorption and vanadium permeability, resulting in better ion conductivity of the membrane [92]. When the GO content in the membrane exceeded the optimal value, the performance of the composite membrane decreased. This is explained by the aggregation of GO on the polymer matrix during the formation of the polymer membrane due to the electrostatic effect of GO nanosheets [35]. Accordingly, they reported that even if the content of GO was as low as 0.01 wt% in the polymer matrix, GO still presented significant agglomeration. To address this issue, GO can also be modified to enhance the performance of the membrane. To mitigate the electrostatic effects and improve the hydrophilicity of GO nanosheets, Ye et al. [35] developed a strategy to utilise in situ grown tungsten trioxide (WO_3_) nanoparticles on the surface of GO nanosheets, which were subsequently introduced into the PFSA matrix to increase ion selectivity of the membrane. Incorporating hydrophilic material such as WO_3_ between or on the surface of GO nanosheets hindered electrostatic binding which was an effective solution to prevent agglomeration. The hydrophilic enhancement of the membrane due to the introduction of WO_3_ on the GO surface solved the issue of low water uptake capacity of the membrane. Furthermore, hydrophilic WO_3_ nanoparticles grown onto the GO nanosheet shortened the proton conduction pathways, thereby increasing the proton conductivity. As a result, under the current density of 120 mA cm^−2^, the hybrid membrane’s cell exhibited higher CE (98.1%) and EE (88.9%) than the Nafion 212.

Similar to CNT and GO, graphitic carbon nitride (g-C_3_N_4_) possesses high surface area and mechanical stability. However, the nitrogen atoms of g-C_3_N_4_ introduce distinct characteristics [93]. A method based on the cross-linking between − NH_2_ groups in g-C_3_N_4_ and − SO_3_H groups in the SPEEK matrix to reduce the vanadium permeation and improve proton transport in VRFB applications was reported [94]. Because of its triangular nanopores and strong hydrophilicity, g-C_3_N_4_ has a great ability to adsorb water, which encourages the creation of a proton channel and raises the IEC value. Like other materials possessing amino groups (–NH_2_/–NH_3_^+^), g-C_3_N_4_ nanosheets may consume numerous –SO_3_H groups in the polymer backbone, which may influence the hydrophilicity of the membrane. Hence, adding sulfonic acid and amino groups to various materials is an effective technique to control the balance between ion permeability and proton conduction, where sulfonic acid groups can offer more proton pathways while amino groups can facilitate proton transportation through the Grotthuss mechanism via electrostatic attraction.

#### Organic Materials

Organic materials are often used to modify IEMs and significantly improve their performance. Triphenylamine (TPAM) is an organic substance that is commonly utilised to raise the IEC value of membranes due to its electron-donating ability. Based on this fact, TPAM was chosen by Quan et al. [95] to modify and dope into SPEEK for VRFB applications. Nitrogen atoms in TPAM can be protonated and used as proton donors, forming hydrogen bonding networks that facilitate proton hopping. In addition, it can effectively interact with the sulfonic acid groups in the polymer matrix to form acid–base pairs, resulting in a decrease in the swelling ratio. Hence, the modified membrane showed a much lower vanadium crossover compared to the pristine membrane. This was considered due to reduction in the water channel size in the presence of TPAM. However, stability tests revealed that the chemical stability of this membrane decreased to some extent. Therefore, the approach using TPAM to improve the performance of IEMs requires further investigation to maintain their stability during operation. Ionic liquids (ILs), commonly known as organic salts in liquid form, consist of cations and anions remaining in a liquid state and are also promising candidates to enhance the overall efficiency of VRFB. These liquids have outstanding thermal stability, a wide electrochemical range, and good ionic conductivity which are highly beneficial in energy storage applications. To capitalise on the benefits of this material, Song et al. [96] introduced 1-butyl-3-methylimidazolium tetrafluoroborate ([BMIm]BF_4_) into poly(oxyphenylene benzimidazole) (OPBI) to fabricate a composite membrane with excellent proton conductivity. [BMIm]BF_4_ contained cations that not only could form hydrogen bonds with the polymer chains but also rejected the vanadium ions permeation through the Donnan exclusion effect. Thus, as the concentration of [BMIm]BF_4_ increased, both vanadium resistance and ionic conduction of the composite membranes also increased. As a result, the single cell using the composite membrane showed better performance compared to the unmodified membrane and Nafion membrane.

**Fig. 7:**
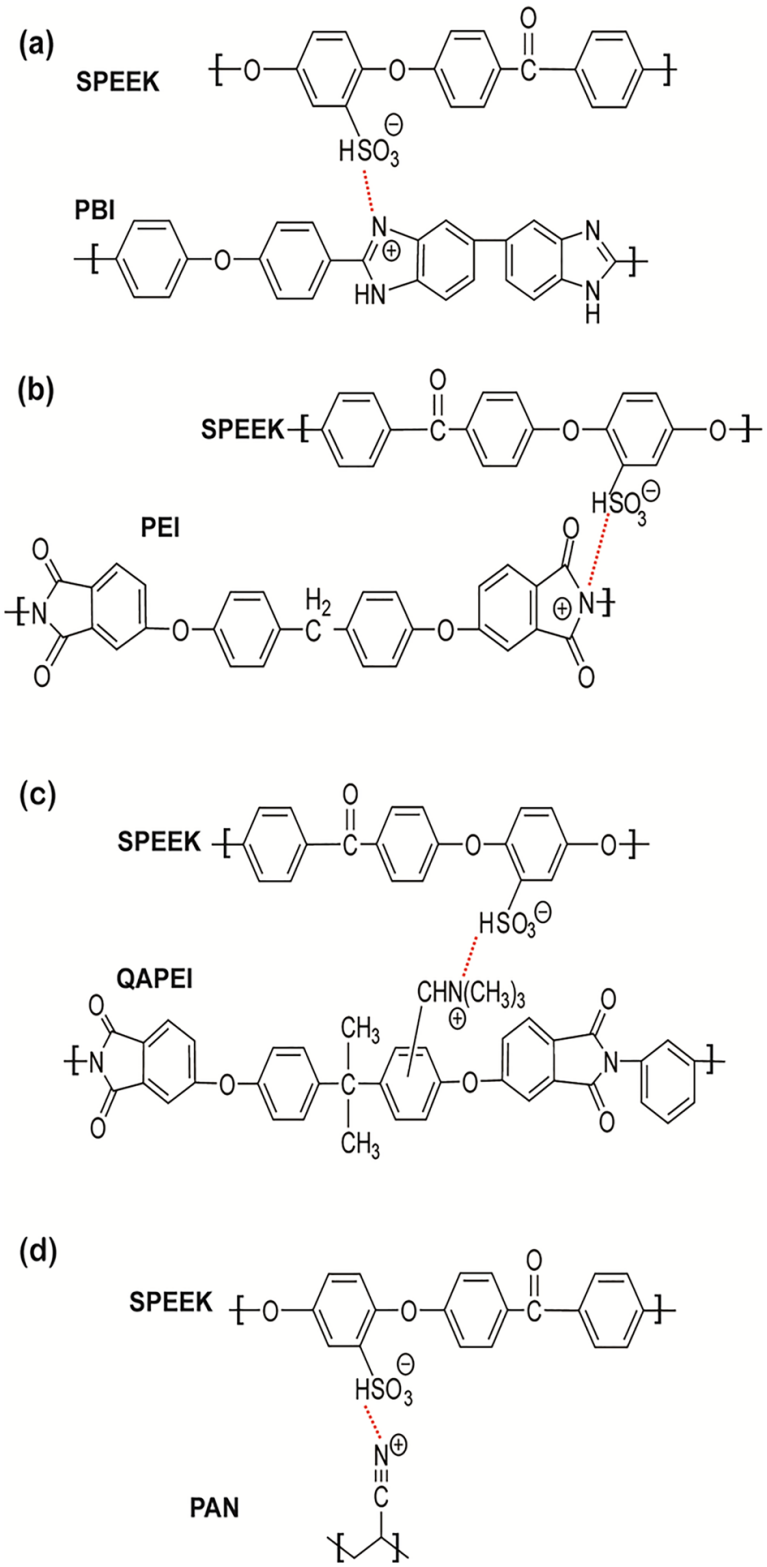
Cross-linking in the composite membrane via acid–base interaction (red dashed line) in polymer blending membranes

Polymers, as a category of organic materials, are commonly used to modify membranes. Polymer co-blending is a common, simple, and efficient approach for membrane modification. The compatibility of the polymer matrix is the key factor for producing homogenous polymer membranes. The strong bonds formed between acidic and basic functional groups in different types of polymers help to enhance the chemical durability and reduce the water channel size of IEMs [97]. The composite with the polymer may reduce the permeability of vanadium ions while simultaneously impeding the proton conductivity of the membrane. Thus, to attain a balance between these two factors, suitable materials and innovative technologies are urgently required. For example, Chen et al. [98] synthesised a SPEEK-PBI blend membrane based on the acid–base interaction between SPEEK and PBI polymer. The conversion of H^+^-SPEEK to Na^+^-SPEEK prevented PBI polymer from precipitating in SPEEK solution, which improved the compatibility of SPEEK and PBI. After that, the internal cross-linking network was successfully induced by the acid–base interaction. As a result, the blended membrane had a uniform and stable morphology, which increased its stability in acidic conditions. In addition, the introduction of PBI led to a denser and thicker membrane, contributing to the reduction of vanadium ion crossover. Meanwhile, proton transportation was still guaranteed by the presence of sulfonated groups and nitrogen groups in the blend membrane. Therefore, the resulting membrane achieved excellent performance during the cell operation (CE: 98.5%, EE: 89.8%) at 80 mA cm^−2^. Many other basic polymers have been blended into the SPEEK polymer matrix, including polyetherimide (PEI) [97], quaternised poly(ether imide) (QAPEI) [99], polyacrylonitrile (PAN) [100] (Fig. 7). The addition of cations from basic polymer to resist vanadium ions was the reason for the reduced vanadium permeabilityof these polymers. Furthermore, the occurrence of acid–base pairs on the surface of blended membranes can enhance their stability and ion selectivity.

In addition to the use of non-fluorinated polymers, fluorinated polymers have also been used to modify membranes to overcome the excessive water absorption, poor chemical and mechanical stability, and other limitations of these membranes [101]. For instance, Ren et al. [101] developed a new kind of AEM by blending quaternized polysulfone (QAPSF) with PVDF. The membrane based on QAPSF has previously demonstrated inadequate mechanical strength that hindered its application in VRFBs [102]. PVDF is a super-hydrophobic polymer with high availability and superior mechanical stability. The QAPSF blended with PVDF could overcome each other’s deficiencies, hence diminishing the vanadium ion permeability and improving the mechanical stability of the membrane. The results proved that the introduction of PVDF can effectively decrease the swelling ratio and permeability of vanadium ion. Thus, cells with the QAPSF/PVDF membrane showed high CE values (> 99%).

Recently, Sadhasivam et al. [103] prepared a novel hybrid membrane for VRFB applications using a homogeneous mixture of alkoxysilane functionalised polymer (ASFP) with Nafion solution. The existence of hydroxyl groups in ASFP limited the availability of free sulfonic acid groups in the Nafion matrix based on cross-linking and/or acid–base reactions. The smaller hydrophilic domain in the membrane contributed to the control of vanadium permeability across the membrane. Overall, the blended membrane showed similar water uptake, IEC, and ionic conductivity but exhibited greater ion selectivity and ultra-low vanadium permeability compared to the pristine membrane.

Green biomaterials, a class of organic matter, are novel candidates for use in composite membrane development that can effectively reduce costs and protect the natural environment [40, 104, 105]. Organic natural materials derived from nature have numerous functional groups, such as hydroxyl (−OH), acidic sulfonate (−SO_3_H) groups, and an extensive intramolecular hydrogen bonding network, which contribute to the excellent proton conductivity of these materials. Cellulose nanocrystal is an environmentally friendly and sustainable nanomaterial mostly sourced from wood pulp, suggesting significant promise as a foundational component for the fabrication of super strong membranes [106]. The basic component of cellulose nanocrystals is glucose which contains many hydroxyl groups in their structure, making them extremely hydrophilic. Furthermore, cellulose nanocrystals are prepared in acidic conditions. Hence, the obtained materials have superior stability when they are embedded in IEMs used for VRFBs. Cellulose molecules can also be modified easily to enhance their interaction with different types of polymer matrix [107]. It is suggested that cellulose can become a suitable material to improve the membrane’s performance for VRFB. Another typical example is lignin, which is also a product made from wood. It is often known as a by-product of the paper industry, which ensures that the supply of this material is abundant and low cost. Ye et al. [40] showed that lignin can be used as an additive in the SPEEK polymer matrix to produce a composite membrane for VRFB applications. Lignin consists of abundant hydroxyl groups that facilitate the transport of protons via an ion exchange mechanism, contributing to the increase in ion selectivity of the membrane. The interaction between −OH and −SO_3_H via hydrogen bonding also increases the membrane’s mechanical stability [108], while lignin can also effectively block the vanadium ions crossover, contributing to better vanadium ion selectivity of the membrane. The battery using SPEEK/lignin membrane exhibited a higher CE (99.5%), EE (83.5%), and longer cycle lifespan at 120 mA cm^−2^.

#### Organic–Inorganic Hybrid Materials

In addition to single type of organic or inorganic material as additive, hybrid materials containing both organic and inorganic materials are often employed to improve the performance of IEMs. One of the most promising approaches to overcome the trade-off of proton conductivity and ion selectivity of IEMs is incorporating metal–organic frameworks (MOFs) in IEMs. MOFs are a distinct group of microporous materials in which multidirectional organic ligands are connected by metal ions or clusters, acting as joints and linkers in the network structure [109]. The large surface area, controllable pore structure, and integrated redox metal core of the pristine MOFs allow them to be used as electrode or electrolyte materials in electrochemical systems. The first study on using MOF as additives in IEMs for enhancing the VRFB performance was published by Liu et al. [110]. They proposed the incorporation of UiO-66, a prototypical Zr-based MOF due to its suitable pore size, strong chemical stability, and ease of functionality. The micropore sizes of UiO-66 are bigger than the size of a proton (0.24 nm) but smaller than the size of a vanadium ion (> 0.6 nm); therefore, the proton can move across the Zr-MOF-loaded membrane while vanadium ions are strictly blocked. However, the lack of functional groups in MOFs limits the proton-conducting ability of the MOF-based membranes. Researches on functionalisation of MOFs have been reported to improve their performance using functional groups such as: −NH_2_ [111] and −OH [110]. Furthermore, Yang et al. [112] loaded phosphotungstic acid (PWA) into the pores of UiO-66-NH_2_ through electrostatic interaction to enhance the proton conductivity of the membrane. PWA is a type of polyoxometalates (POMs) that is a strong acid and thermodynamically stable. POMs are a potential material that can be utilised to fabricate high proton-conductive membranes since the protons in them can form hydrogen bonds with water, forming H_3_O^+^ or H_5_O_2_^+^. By introducing UiO-66-NH_2_@PWA into the polymer matrix, the ion-conducting domains of Nafion membranes were successfully restricted, contributing to the suppression of vanadium ion penetration. The VRFBs using the Nafion-(UiO-66-NH_2_@PWA) showed lower vanadium permeability (3.46 × 10^−7^ cm^2^ min^−1^) and better proton conductivity (0.092 S cm^−1^), which then led to superior ion selectivity (2.66 × 10^5^ S min cm^−3^). Nevertheless, a serious problem occurred owing to the leakage of PWA from the membrane during the cell operation. It was attributed to the weak electrostatic interactions between PWA and MOFs that were not sufficiently strong to retain PWA within the pores of MOF under the aqueous solution. Zhai et al. [113] used a novel technique to avoid PWA leakage by chemically attaching PWA with MIL-101-NH_2_ MOF through a sintering process. As a result, numerous pores of MIL-101-NH_2_ were occupied by PWA, which benefited the ion selectivity of IEMs, as it could both provide a barrier to prevent vanadium from penetrating and offer extra proton hopping sites to help the transportation of proton. Because of the strong electrostatic attractions between the hydrophilic groups in PWA/MIL-101-NH_2_ and the polymer matrix, as well as its excellent stability in acidic conditions, the composite membrane had a better performance with high thermal and chemical stability. Table 1 summarises some key parameters of the modified IEMs.

**Table 1:** Summary parameters of the modified IEMs

Polymer matrix	Material & Method	Conductivity (mS cm^−1^)	Permeability (10^−7^ cm^2^min^–1^)	Current density (mA cm^−2^)	CE (%)	EE (%)	Ref.
Nafion 117	SiO_2_ (in-situ sol–gel)	56.2	3.8	40	90.2	85.6	[63]
Nafion 117	Si/TiO_2_ (in-situ sol–gel)	–	4.3	30	94.8	77.9	[64]
Nafion 212	Lignin (blending)	16	0.85	50	92.7	86.3	[66]
Nafion	SPEEK (blending)	–	1.928	50	97.6	83.3	[67]
SPAE	Partially fluorinated SPAE	61	0.075	50	97.5	93.4	[74]
SPPEK	PWA (blending)	19.04	5.75	60	98.75	74.58	[76]
Fumasep	SiO_2_ (in-situ sol–gel)	–	4.24	40	92	73	[81]
FAP SPEEK	Sulfonated TiO_2_ (blending)	61	8.55	50	95.3	82.3	[82]
SPEEK	Sulfonated TiO_2_ (blending)	23.2	7	100	98.2	80.2	[83]
SPAEK	Ce_2_Zr_2_O_7_ (blending)	77	0.0116	40	99.3	82.7	[84]
Nafion 212	TiO_2_ nanotube (blending)	12.5	1.15	120	97.7	81.4	[85]
SPI	MoS_2_ nanosheet (blending)	50.4	0.65	20	99	80	[87]
SPI	SiC (blending)	–	0.88	20	99	84.4	[88]
SPEEK	CNT (electrospinning)	42	0.67	100	98.1	86.2	[89]
Nafion 117	GO (blending)	35	5	100	91.5	78.8	[92]
PFSA	PTFE/WO_3_@GO	17	3	120	98.1	88.9	[35]
SPEEK	g-C_3_N_4_	7.9	3	30	97	83.6	[93]
SPEEK	TPAM	70	3.04	60	97.5	83.8	[95]
OPBI	BF_4_ ionic liquid (blending)	7.4	0.0525	40	99.3	92.4	[96]
SPEEK	PEI (blending)	65	0.23	50	93.9	79.8	[97]
SPEEK	PBI (blending)	–	–	80	98.5	89.8	[98]
SPEEK	QAPEI (blending)	47.4	1.3	50	96.1	88.5	[99]
SPEEK	PAN (blending)	15	11.3	80	96.2	83.5	[100]
Nafion	ASFP (blending)	68	1.26	80	96.4	76	[103]
SPI	Sulfonated methylcellulose (blending)	43.6	1.75	100	99.2	68	[107]
SPEEK	Lignin (blending)	29.6	0.17	120	99.5	83.5	[40]
SPEEK	UiO-66 (blending)	30	2.4	50	98.9	91.4	[110]
Nafion	UiO-66-NH_2_@PWA (blending)	92	3.46	100	93.3	72	[112]
SPEEK	S-UiO (blending)	70	0.8	120	99	83.9	[111]
SPEEK	MIL-101-NH_2_@PWA (blending)	70	1.4	120	99	82.1	[113]

### Amphoteric Ion Exchange Membranes

**Fig. 8:**
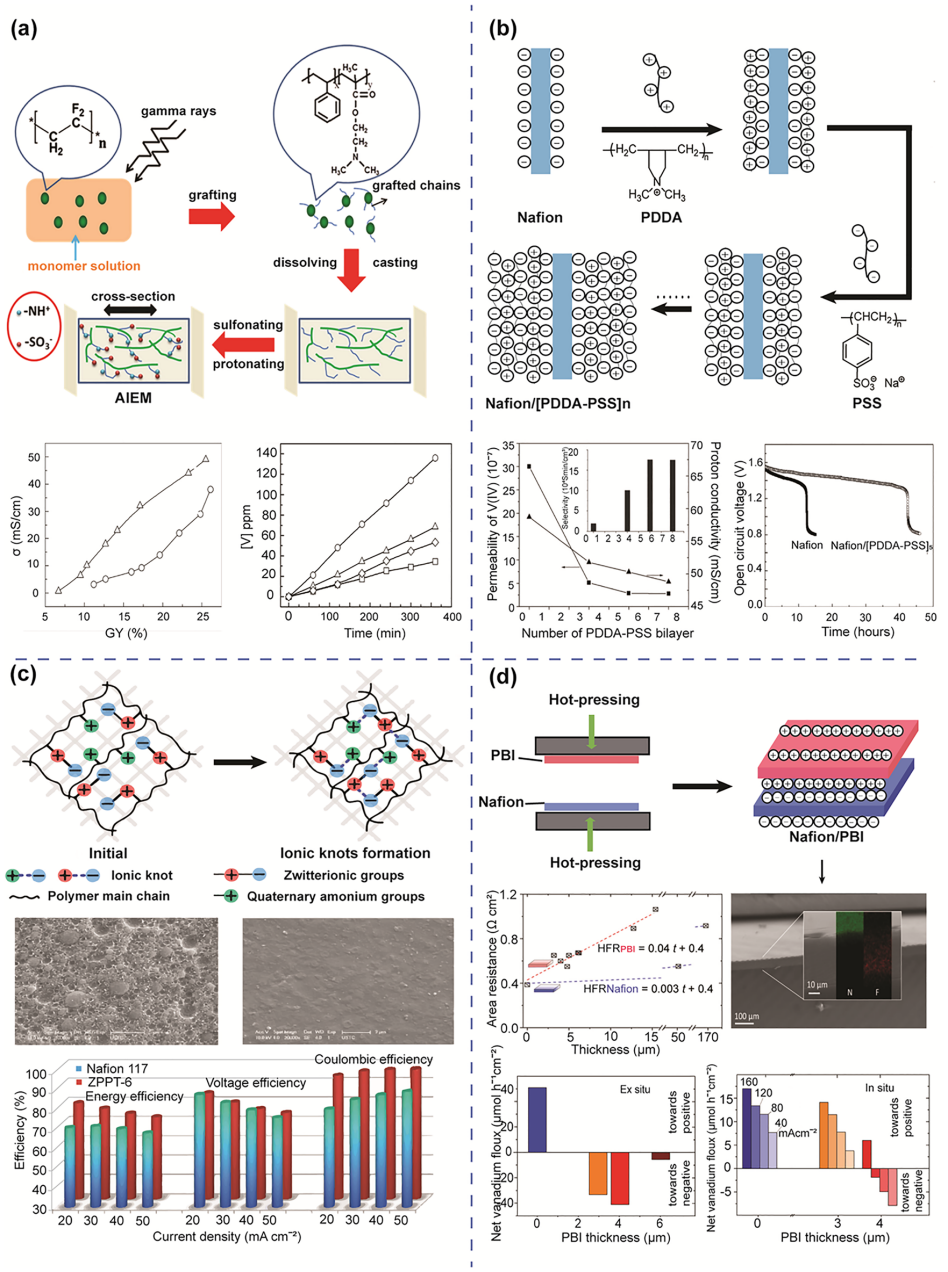
**a** Schematic illustration of the AIEM using the grafting method, ion conductivity, and permeability of AIEM, reproduced from Ref. [116] with permission from Elsevier, copyright 2012. **b** Preparation of Nafion–[PDDA-PSS]_n_ membrane through layer-by-layer self-assembly technique, permeability, open circuit, and performance of battery cell using Nafion–[PDDA-PSS]_n_ membrane, reproduce from Ref. [117] with permission from Royal Society of Chemistry (RSC), copyright 2008. **c** The ionic knots produced by zwitterionic groups via in situ polymerisation, SEM images of cross section and surface of the membrane, the comparison of overall performance between Nafion 117 and modified membrane under different densities, reproduced from Ref. [121] with permission from Elsevier, copyright 2015. **d** The AIEM using hot-pressing method, cross-sectional analysis of a PBI/Nafion bilayer membrane, area resistance and permeability of bilayer membrane, reproduced from Ref. [123] with permission from WILEY–VCH, copyright 2019

The amphoteric ion exchange membranes (AIEMs) contain both cationic and anionic functional groups on the polymer backbone. AIEMs possess all properties of AEMs and CEMs including high ion conductivity, superior chemical strength from CEMs as well as a limited vanadium crossover rate from AEMs. Therefore, the percentage of cation and anion functional groups in AIEMs will determine the characteristics of the membrane. In the past decades, methods to introduce functional groups into AEMs and CEMs have been reported. Skyllas-Kazacos et al. [114] first modified the Selemion AMV anion exchange membrane by grafting sulfonic acid onto the polymer matrix. The presence of -SO_3_H groups improved the ionic conductivity of the membrane addressing the weakness of AEMs. More recently, Qui and colleagues prepared an AIEM using styrene and dimethylaminoethyl methacrylate (DMAEMA) that were grafted onto the PVDF backbone through an irradiation grafting method [115]. The higher grafting yield resulted in higher ion conductivity while the vanadium permeability decreased with the increase of DMAEMA concentration. The grafting method can be integrated with other techniques such as solgel and solution phase inversion to produce various AIEMs. For instance, to further improve the modification method, Ma et al. [116] combined the radiation grafting method with the solution phase inversion technique to provide the AIEMs (Fig. 8a). The membranes produced through the new method exhibit a slightly elevated water absorption capacity, resulting in higher ion conductivity and reduced local resistance. Moreover, the composition of the AIEMs is also easily adjusted by controlling the initial monomer fraction, increasing its versatility in VRFB applications.

Besides the grafting method, there are several techniques to modify and fabricate AIEMs, such as surface coating, layer-by-layer assembly, interfacial polymerisation, or hot-pressing. Xi et al. [117] applied a layer-by-layer self-assembly method to prepare an AIEM by alternating layers of poly(diallyldimethylammonium chloride) (PDDA) and polyanion poly(sodium styrene sulfonate) (PSS) onto the surface of a Nafion membrane (Fig. 8b). The layer-by-layer assembly enables fabrication of multilayer membranes with controllable structures and compositions by the alternating adsorption of oppositely charged ions onto the surface of the substrate via electrostatic interaction. [118]. Due to the formation of the PDDA-PSS multilayer, it effectively blocks the ion transfer path and reduces the permeation of vanadium ions. As expected, the modified membranes demonstrated a high CE (97.6%) and EE (83.9%), along with a lower self-discharge rate compared to the Nafion 117 membrane.

The interfacial polymerisation method is widely used in the synthesis of thin-film composite membranes to form a stable layer on the surface of the membrane [46]. Wang et al. [119] synthesised novel AIEMs by direct polymerisation of pendant quaternary ammonium groups onto the sulfonated poly(fluorenyl ether ketone) (SPFEK). The presence of quaternary ammonium groups accelerates the Donnan exclusion effect, resulting in decreased vanadium ion permeation in AIEMs, while maintaining stability and proton conductivity of the original SPFEK membrane. Meanwhile, Yan et al. [120] combined SPEEK with imidazolium-functionalised polysulfone (ImPSf) to prepare several AIEMs. The addition of the ImPSf successfully limits the transport of vanadium ions across the membrane thanks to the Donnan exclusion effect and the ionic cross-linked connections between the imidazolium and sulfonic groups. As a result, the membrane delivered better CE and EE than the bare Nafion membrane at a high current density. Recently, a novel quaternised membrane bearing with zwitterionic groups ([CH_2_N^+^(CH_3_)_2_CH_2_CH_2_CH_2_SO_3_^−^]) using a solvent-free approach was reported to reduce the vanadium permeability [121]. A zwitterionic polymer containing both positively and negatively charged functional groups within the same monomer unit showed a substantial dipole moment and excellent stability in a broad temperature range [121]. The mechanical strength of the membrane was enhanced significantly due to the strong chain-chain interactions between the quaternary ammonium groups and the zwitterionic groups (Fig. 8c). The existence of the copolymer also greatly enhances the chain packing density, which greatly improves membrane stability and reduces vanadium ion permeability.

Polymer blending is also a popular method to fabricate AIEMs. For instance, Liu et al. [99] directly blended quaternised poly(ether imide) (QAPEI) onto the SPEEK polymer matrix to form a novel AIEM. This membrane produced a larger extent of phase separation, which was advantageous for proton transportation. The introduction of QAPEI also decreased the water uptake and swelling ratio, reducing permeability of vanadium ion crossover and improving ion selectivity. Compared to commercial Nafion membranes, the AIEM membranes demonstrated remarkable performance, resulting in a high CE of 96.1% and EE of 88.5%. Liao et al. [122] prepared a desirable AIEM for VRFB by incorporating benzimidazole units with controlled content into fluoro-methyl sulfonated poly(arylene ether ketone). Within the AIEM, a dense structure was formed through ionic cross-linking via acid–base interactions, supported by covalent cross-linking achieved through the incorporation of p-xylylene dichloride as a cross-linker. The denser membrane is significantly useful in protecting the main chains from the attack of oxidised species. In addition, the ionic cross-linking between the acid and basic moieties, or covalent cross-linking, compacted the polymer matrix, reduces the water uptake and swelling ratio, resulting in narrower channels and inhibiting the transfer of hydrated vanadium ions across the membrane. Hence, the modified membrane showed outstanding performance with CE (99%), EE (89%), and VE (90.7%) at 30 mA cm^−2^.

The hot-pressing method involves the application of heat and pressure to bond layers of materials together is commonly used to promote the adhesion between different layers of a membrane to enhance the overall structural integrity of the composite membrane. Recently, Oldenburg et al. [123] used hot-press method to make an amphoteric PBI/Nafion bilayer membrane (Fig. 8d). By combining mPBI with the Nafion membrane to balance the cationic and anionic exchange groups in the AIEM, the net vanadium flow and the capacity fading were suppressed. The result showed that the overall performance of a VRFB using a bilayer membrane was improved compared to the original Nafion membrane at the same current density.

### Porous Membranes

**Fig. 9:**
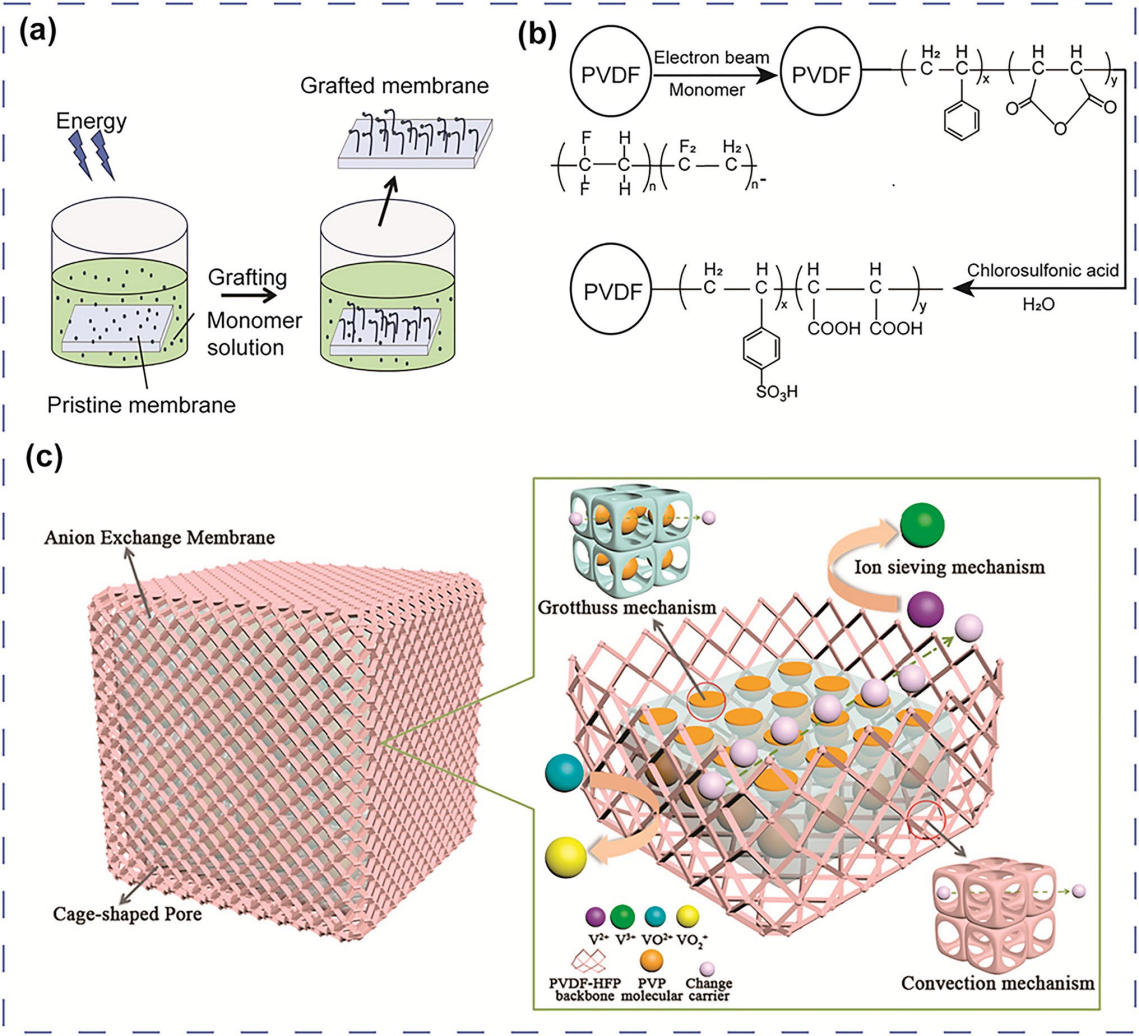
**a** Schematic process of the composite membrane synthesis using grafting method. **b** Synthesis route for the preparation of PVDF-*g*-PSSA-*co*-PMAc ion exchange membranes [129]. **c** The ion transport mechanism and vanadium ion permeation process in PVDF/HFP membrane, reproduced from Ref. [132] with permission from Elsevier, copyright 2019

Differing from IEMs, the ion transport mechanism of porous membranes primarily relies on the physical structure and properties of the pores rather than fixed charged groups. Without functional groups, protons are exchanged through the pores, whereas vanadium ions are excluded due to the pore size exclusion effect [42]. Compared to IEMs, porous membranes possess greater chemical and mechanical stability and are considered a promising membrane for VRFBs. Various low-cost porous membranes have been reported, including PVDF [124], PES (polysulfone) [125], PVC (polyvinyl chloride) [48], and PBI [126]. Because of the hydrophobic backbone of these polymers, creating considerable porosity is essential to facilitate ion transport in the materials. The porosity provides the necessary channels for ion movement, allowing for enhanced proton conductivity while maintaining the mechanical and functional properties of the membranes. Nevertheless, excessive porosity gives rise to the possibility of forming penetrative pores, which would lead to inadequate ion selectivity. As mentioned above, a reduction in pore size also affects the ion transport mechanism through the membrane. Therefore, more modification techniques and investigations are needed to satisfy the pore size exclusion criteria to enhance the ion selectivity of porous membranes. PVDF is one of the most widely used materials in porous membranes due to its excellent chemical resistance and low cost [127]. However, because of the high hydrophobicity of the polymer matrix and high permeability caused by the microporous structure, improving the compatibility between the electrolyte and a PVDF membrane is highly important. Pore filling with hydrophilic materials is a possible method to enhance the ion conductivity as well as reduce the pore size of the porous membrane. Cao et al. [128] fabricated a hydrophilic porous membrane by grafting polyvinylpyrrolidone (PVP) on the PVDF matrix via grafting polymerisation and cross-linking reaction. Meanwhile, Qiu et al. [129] chose styrene and maleic anhydride to graft onto PVDF (Fig. 9a, b). The grafted membrane was then sulfonated and hydrolysed to produce an IEM (PVDF-*g*-PSSA-*co*-PMAc) used for VRFBs. Later, Zhou et al. [130] also chose styrene monomer and BaTiO_3_ powder to graft onto the PVDF membrane. Adding additives or functional groups to the PVDF is also an efficient method to enhance the properties of porous membranes. For instance, by the introduction of glycidyl methacrylate (GMA), Tian et al. [131] modified the PDVF into an AEM. The modified membranes successfully reduce the vanadium permeability of the membranes by limiting the ion transport channels; however, the ion conductivity also decreases as a result of increase of the area resistance. New fabrication processes have been developed recently to achieve balanced vanadium permeability and ionic conductivity. Lu et al. [132] fabricated a novel porous AEM with a core–shell structure composed of poly(vinylidene fluoride-hexafluoropropylene) (PVDF-HFP) and PVP as the core and VO_2_^+^ as the shell (Fig. 9c). The combination of the pore size exclusion effect and the Donnan exclusion effect prevents vanadium ion permeation, ensuring excellent ion selectivity of the membrane with exceptional chemical stability. Moreover, amine groups of PVP and the cage-shaped pores on the surface and inside the membrane contribute to the high ion conductivity of the porous membrane. As a result, a great balance between high ion selectivity and high ion conductivity was achieved, thus leading to a high CE (98.16%) and EE (88.01%) at 80 mA cm^−2^.

Aside from PVDF, a variety of other polymers have been commonly used to fabricate porous membranes for VRFB systems currently. The majority of research concentrated on combining diverse materials using different techniques to improve membrane properties. Xi et al. [133] fabricated PES/silica composite porous membranes using the solgel method to control the size of pore and the pore size distribution. Exploiting the different affinities between PES and different solvents, the filler content was controlled by adjusting the swelling degree. After silica modification, the pore size was reduced owing to filling of the pores with the silica gels. The single cell assembled with the silica-modified membrane exhibited significantly superior performance (CE: 97%, EE: 83%) compared to the original porous membrane (CE: 86%, EE: 76%). Nevertheless, the excessive addition of modification materials into the porous matrix can destroy the structure of the porous membrane to a certain extent. When the mass fraction between the added material and the polymer matrix is too high, it provides a high elongation, causing the break of the membrane. [134]. In 2016, by using the acid doping method, Yuan and colleagues successfully filled the pores of the PBI matrix with a sulfonic acid solution [135]. The modified PBI membrane exhibited a porous structure with thousands of micron-sized voids, wherein sulfonic groups maintained high proton conductivity, whereas the positively charged groups of the PBI matrix generated multiple blockages for the vanadium ions, hence preserving high conductivity. Unlike inorganic fillers, which can leach out over time or under harsh conditions, acid-doped membranes retained their functional groups better, leading to improved durability in long-term applications. Furthermore, acid doping created consistent, distributed proton-conducting sites within the polymer matrix, leading to more uniform conductivity across the membrane. Accordingly, the acid-doped PBI membrane demonstrated a superior capacity retention over 1300 cycles.

Blending with polymer is a highly effective method to adjust the pore size of the membrane [136]. Mixing two or more polymers leads to an increased concentration of polymer solution, then raises its viscosity. Hence, the mixing process was slower, making the membrane thicker and the pore size smaller. For instance, Luo et al. [137] mixed nanoporous PBI polymer with porous PBI membrane to make a hybrid nanoporous membrane with a reduced vanadium crossover rate and low cost. Wu et al. [138] developed a novel polysulfone-polyvinylpyrrolidone (PSF/PVP) membrane displaying superior ion selectivity, exceptional chemical stability, and cost-effectiveness for VRFB systems using a simple polymer blending method. They further restricted the crossover of vanadium ions by loading GO nanosheets onto the PSP/PVP porous membrane [139], resulted in a reduction of both proton conductivity and vanadium ion permeability.

**Fig. 10:**
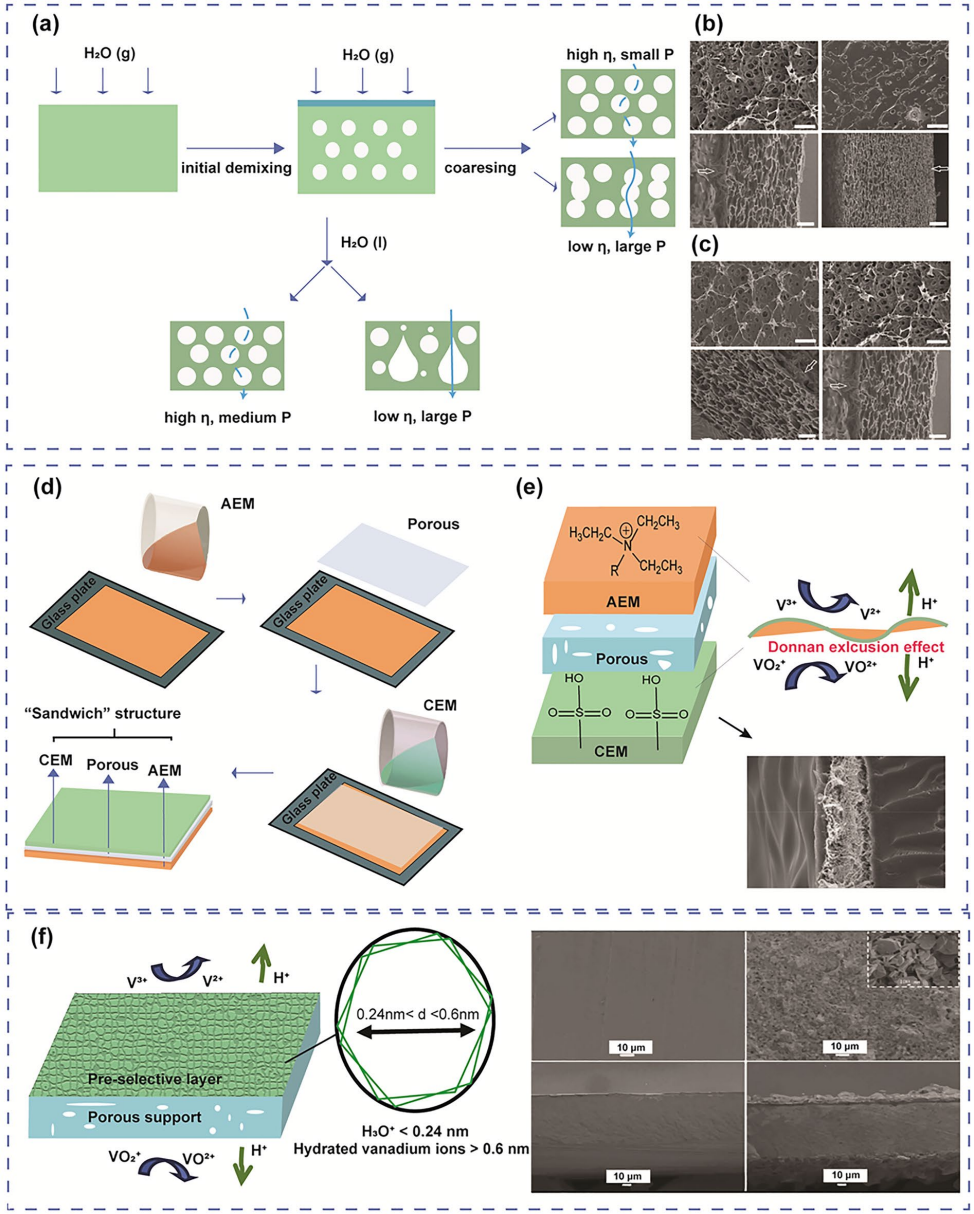
**a** Illustration of the vapour-induced phase separation technique, η is the viscosity of the polymer-water system, and P is the vanadium permeability of the resulting membranes, the surface (upper) and cross-sectional (lower) FESEM images of membranes using vapoured induced phase separation process with **b** different concentration of polymer solution, **c** different relative humidity, reproduced from Ref. [141] with permission from Elsevier, copyright 2018. **d** Process for the preparation of bipolar membranes; **e** design principles of the bipolar membrane with sandwiched structure, SEM image of cross section of the bipolar membrane, reproduced from Ref. [144] with permission from Elsevier, copyright 2018. **f** Design principle of porous membrane bearing a zeolite flake layer, surface, and cross-section morphology of a pristine membrane (left) and a zeolite-coated membrane (right), reproduced from Ref. [145] with permission from WILEY–VCH, copyright 2016

Sizov et al. [140] developed a novel fabrication method to reduce the pore size of the membrane using a supercritical CO_2_-assisted phase inversion approach while Luo and colleagues [141] applied a phase separation method induced by water vapour to prepare the poly(2,5-benzimidazole) (ABPBI) porous membrane. The normal phase inversion method refers to the controlled transformation of cast polymer film from a liquid state to a solid state [142]. During the phase inversion process, the homogeneous polymer solution film was in a thermodynamic equilibrium state. This state was then broken due to the sudden change in solvent and non-solvent. Consequently, the polymer solution was separated into two phases, including the polymer-rich phase (coagulated phase) and the polymer-lean phase (dilute phase). The coagulated phase formed the solid membrane while the dilute phase was removed to form pores within the membrane. The properties of the membranes (porous, dense, or composite) depend on the specific conditions during phase inversion. The phase inversion approach using CO_2_ assistance produced an increase in the concentration of CO_2_ inside the polymer matrix rather than the solvent, which resulted in the solid–fluid phase separation and the formation of the fibrous structure. Pores can appear as a result of the nucleation and expansion of CO_2_ gas bubbles during a system’s depressurisation, i.e. in a process called foaming [143]. Faster depressurisation increased the nucleation rate and density and stopped the cells from gathering, which increased the quantity of smaller-sized pores. The increased porosity leads to slightly higher proton conductivity but still maintains a stable level of vanadium permeability. Meanwhile, in the phase separation method involving polymer solutions and non-solvents, the thermodynamic and kinetic aspects of the interaction between the polymer, solvent, and water are crucial in shaping the characteristics of porous membranes. During water vapour-induced phase separation, a liquid layer is developed on the membrane surface, which hinders the phase separation process within the membrane bulk. This in turn mitigates vanadium ions permeation of the modified membranes. For example, in the vapour-induced phase separation process, while the polymer-water system remains in contact with water vapour (high solution viscosity), the polymer-lean phase will undergo further coarsening, leading to the formation of macropores with poor connections and thicker polymer layers between the macropores [141]. As a result, the membrane exhibited limited permeability to vanadium ions. In contrast, when the system is isolated from water vapour (low solution viscosity), the macropores with enhanced interconnectivity and porous polymer layers between the macropores were formed, which led to higher vanadium ion permeability (Fig. 10a-c).

Surface coating is one of the favourable ways to achieve high ion selectivity with the membranes. This method can be applied to both IEMs and porous membranes. By coating a barrier layer on the surface of the membranes, constant barrier is created to limit the vanadium ions permeation and to enhance the membrane’s chemical stability. Due to this unique composition, these membranes are typically represented by a sandwich structure. For example, a sandwiched bipolar membrane was prepared using porous polytetrafluoroethylene (PTFE) reinforced QAPSF and SPEEK (Fig. 10d). Each layer had different functions in the membrane. The positively charged layer prevented the vanadium ions from travelling through the membrane via the Donnan exclusion effect while the negatively charged layer facilitated the transportation of protons through the membrane. The porous layer in the middle can enhance the mechanical as well as chemical stability of the bipolar membrane. Overall, this bipolar membrane had a relatively low vanadium permeability. However, the proton conductivity is low in general because of hinderance of the proton transport by the positively charged layer to some extent.

Besides polymers, other inorganic materials are also useful to modify porous membranes. Nanomaterials such as zeolites [145] and boron nitride [146] can be coated on the surface of porous membranes to form a “pre-selective layer”, improving ion selectivity of the membranes (Fig. 10e). The zeolite frameworks contain a significant quantity of exchange cations (Na^+^) present at AlO_4_^−^ that are advantageous for exchanging with H^+^, thereby providing excellent proton conductivity. In addition, the zeolitic pores remain relatively large enough to ensure transport of the proton while still blocking vanadium ions across the membrane. Porous boron nitride with high nanoporosity and stability is also an attractive material additive for membrane development. Using Nafion resin as connectors and a source of ion exchange groups, a unique nanoporous boron nitride bifunctional ion-selective layer was sprayed onto the PEI membrane to create a highly efficient double-layer membrane [146]. Accordingly, the membrane demonstrated strong ionic conductivity and selectivity over long-term cycling performance (700 cycles). Mögelin et al. [147] prepared porous glass membranes with reduced pore sizes using a combination of SiO_2_, B_2_O_3_, and Na_2_CO_3_ at high temperatures. This membrane possessed superior chemical, thermal, and mechanical stability attributable to the distinctive properties of glass. Nevertheless, the area resistance of glass membranes far exceeds that of conventional membranes, which needs to be further reduced for VRFB applications.

Recently, polymers of intrinsic microporosity (PIMs) have been regarded as a novel class of ion transport membranes [24]. PIMs have a rigid structure with interconnected micropores of less than 2 nm, which are suitable for use as an ion-selective membrane in VRFB applications. This type of membrane has been applied in RFBs with high ion conductivity and long-term stability [24]. It has shown that PIM materials have great potential for RFBs due to their porous structure and ease of modification. For instance, Xia et al. introduced PIM material into SPEEK matrix to prepare a high-performance blend membrane [148]. The results indicated that the incorporation of this material could effectively enhance the drawbacks of pure SPEEK membrane, which are low vanadium resistance and poor chemical stability. The CE and EE of the cell using the blend membrane is enhanced significantly (99% and 83%) compared to that of the cell with SPEEK membrane (96% and 74%) under the current density of 120 mA cm^−2^. Currently, Chu and colleagues developed and produced a series of intrinsic microporous sulfonated polyimides with varying surface areas and sulfonic groups through the copolymerisation of 4,4′-(hexafluoroisopropylidene) diphthalic anhydride (6FDA) with 3,5-diamino-2,4,6-trimethylbenzenesulfonic acid (TrMSA) and 2,4,6-trimethyl-1,3-phenylenediamine (DAM) utilising a basic one-step polymerisation method [149]. Overall, the performance of these intrinsic microporous membranes was better than that of the commercial Nafion membrane. Nevertheless, in harsh conditions, physical degradation caused by molecular relaxation present a serious threat to the long-term performance of PIMs membranes for VRFB applications [150]. As a result, most of the PIM-based membranes had low-capacity retention compared to other types of IEM.

**Fig. 11:**
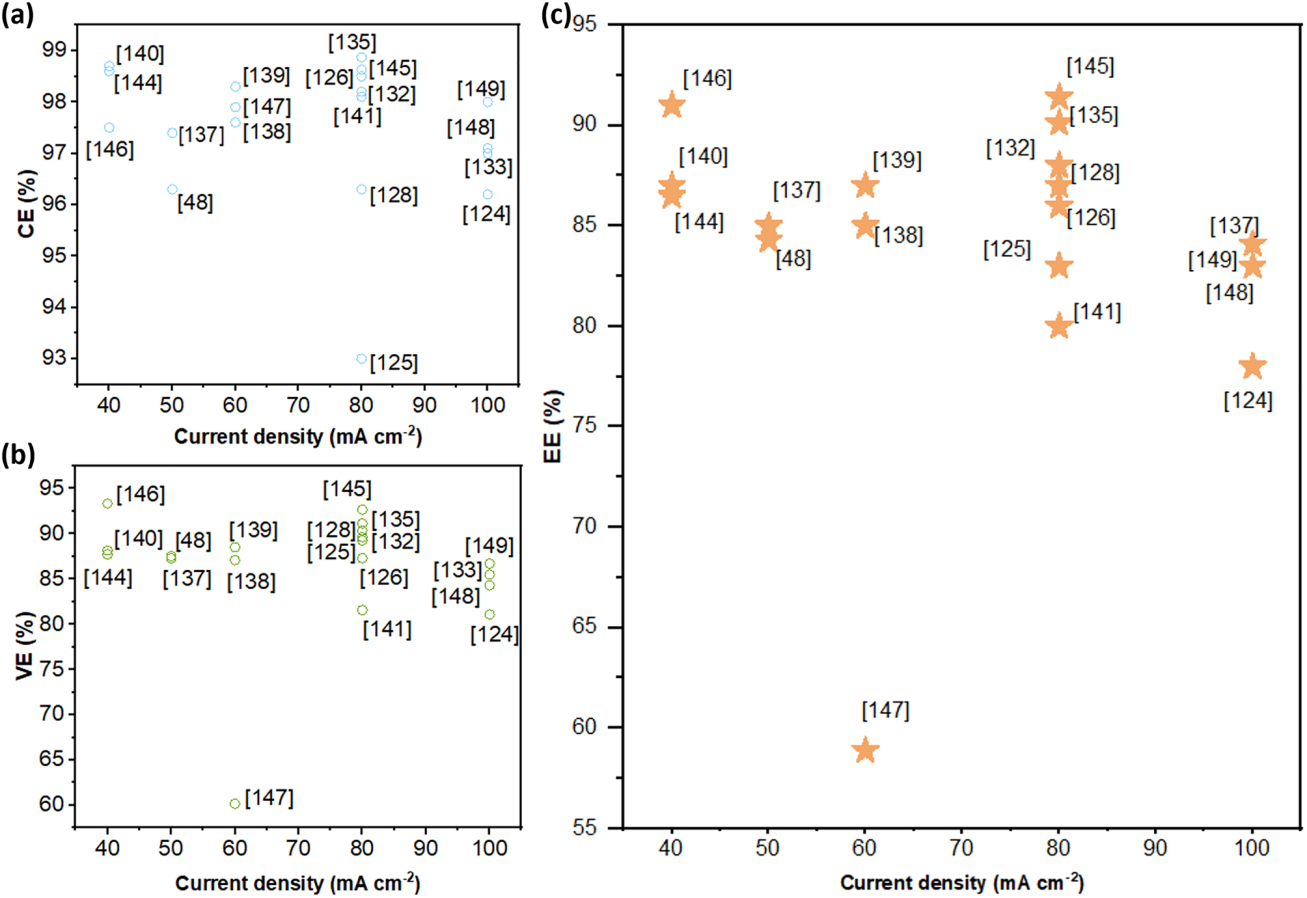
Summary of **a** CE, **b** VE, and **c** EE comparison of various modified porous membranes, reproduced from Refs. [48, 124–126, 128, 132, 133, 135, 137–141, 144–149]

 Figure 11 compares the overall performance of various porous membranes, including CE, VE, and EE. Although the porous membrane represents a viable option for broadening choices in materials compared to IEM, the high area resistance remains a critical challenge, leaving a low VE. Furthermore, the porosity may cause lower mechanical strength as well as the potential of pore fouling, where suspended particles or degradation products from the electrolyte, electrode, or membrane can accumulate within the pores, leading to blockage and a reduction in ion transport efficiency [151]. As a result, porous membranes have been used as a fundamental layer for attaching ionic groups or as an additional layer for IEM, allowing integration of the beneficial features of several membrane types.

### Ion-Solvating Membranes (ISMs)

**Fig. 12:**
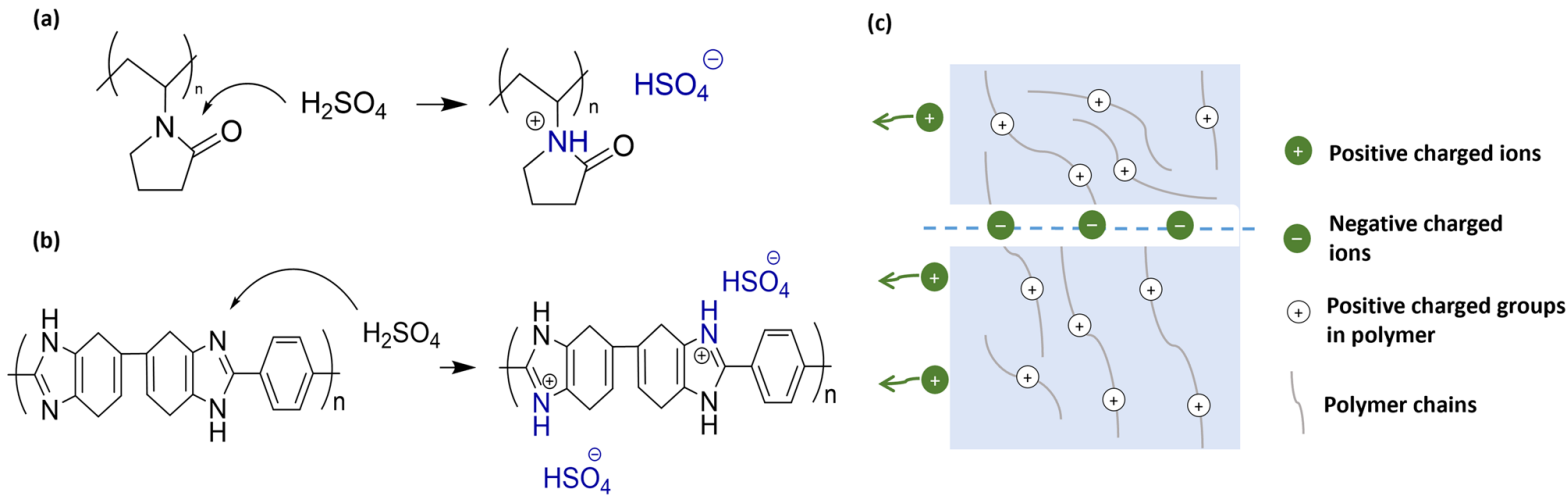
Interaction of **a** PVP and **b** PBI with the supporting electrolyte (H_2_SO_4_). **c** Ion transport mechanism in ion-solvating membranes

Ion-solvating membranes are considered to provide a new approach for ion transport compared to ion-conducting membranes. In ISMs, ion transport typically occurs through solvation coordination with functional groups in the membrane matrix, often via a hopping mechanism rather than traditional ion exchange or diffusion alone (Fig. 12a-c) [152]. ISMs include fundamental heterocyclic rings that can interact with supporting electrolytes to form a homogeneous network, thus facilitating ion conduction [153]. Some typical examples of ISMs include: polybenzimidazole (PBI) [154], polyvinylpyrrolidone (PVP) [47], and poly(p-terphenyl-co-acetylpyridine) (PTAP) [155]. The ion conductivity mechanism in ISMs is influenced by the polymer chain mobility, amorphous regions in the polymer, as well as the heteroatom-cation interaction [156]. In ISMs, despite the absence of ionic groups, nitrogen atoms in polymer chains may function as a proton acceptor that can interact with sulfuric acid through hydrogen bonding or acid–base complexes, allowing the polymer backbone to be positively charged. PVP was the first polymer material to fabricate ISMs for VRFBs. Zhang et al. [157] proposed the PVP-based membrane by using a homogeneous blend mixture of poly(ether sulfone) (PES) with PVP. The ionic conductivity of ISMs is mostly independent of the preparation conditions but is determined by the concentration of supporting electrolytes during the post-treatment process. The blended membrane was subjected to post-treatment with sulfuric acid to increase hydrophilic sites in the polymer matrix that create pathways for hydrated ions. The formation of positively charged quaternary imidazolium groups also results in charge repulsion with positively charged vanadium ions through the Donnan exclusion effect, decreasing the amount of vanadium ions across the membrane. The results demonstrated a comparable effect when the blended membrane effectively suppressed vanadium ion crossover with 99% CE. However, the high hydrophilic characteristics of the blended membrane led to the leakage of PVP during battery operation. To solve this problem, Zeng et al. [158] prepared polyvinylpyrrolidone-based semi-interpenetrating polymer networks by using a photo-induced cross-linking process between PVP and 4, 4′-diazido-2, 2′-stilbenedisulfonic acid disodium salt. A well-established hydrophilic/hydrophobic microstructure was formed, reducing the swelling behaviour and vanadium permeability of the membrane. Thus, this membrane showed better chemical stability with a slower degradation time while maintaining satisfactory performance.

PBI and its derivatives are also widely used in ISMs due to their combination of porous and dense characteristics. In contrast to PVP, PBI possesses superior chemical and mechanical stability that allows it to be used directly as the membrane in VRFB. Zhou et al. [154] utilised meta-type PBI (mPBI) membranes in VRFBs to reduce the crossover of vanadium ions and capacity loss owing to reduced ionic channel size and the positive charge on the PBI backbone. However, the ion transport of mPBI is still limited, leading to low ion conductivity at high current density. To solve this problem, Jang et al. [159] developed H_2_SO_4_-doped dense polybenzimidazole (PBI) membrane that contains poly[2,2′-(2-benzimidazole-*p*-phenylene)-5,5′-bibenzimidazole] (BI*p*PBI). These membranes have a completely amorphous structure, which provides extra benzimidazole side groups, resulting in increased absorption of both acid and water. The improvement of acid and water uptake reduces the area resistance and increases the ion transportation rate. The result showed that the single cell using the BI*p*PBI membrane had much better EEs (78%–95%) compared to those of the unmodified membrane (66%–90%) at 20–100 mA cm^−2^. Another effective technique to increase the proton conductivity of mPBI is H_3_PO_4_-assisted pre-swelling [160]. The pre-swelling approach generates a higher H_2_SO_4_ doping level, thereby significantly lowering the areal resistance of the mPBI membrane. Meanwhile, Xia et al. [161] synthesised a series of highly sulfonated PBI copolymers that were covalently cross-linked by an epoxy resin using pendant amino groups. The coexistence of grafted sulfonic acid groups and doped acid creates a synergistic environment for ion transport, resulting in extremely high ion conductivity. Moreover, the cross-linker produces superior mechanical properties and enhances the vanadium ion barrier inside the membrane. As a result, the modified PBI membrane showed 3–4 orders of magnitude lower ion permeability than that of the Nafion membrane. In general, ISMs possess excellent ion conductivity based on their well-structured pathways while maintaining good solvation of the ions. However, it may increase permeability of undesired vanadium ions, reducing the ion selectivity of ISMs. In addition, the long cycling performance of ISMs in VRFB cells is also not fully studied in the literature. In short, increasing ion selectivity and chemical stability are still the critical challenges of ISMs for large-scale VRFBs.

## Summary

**Fig. 13:**
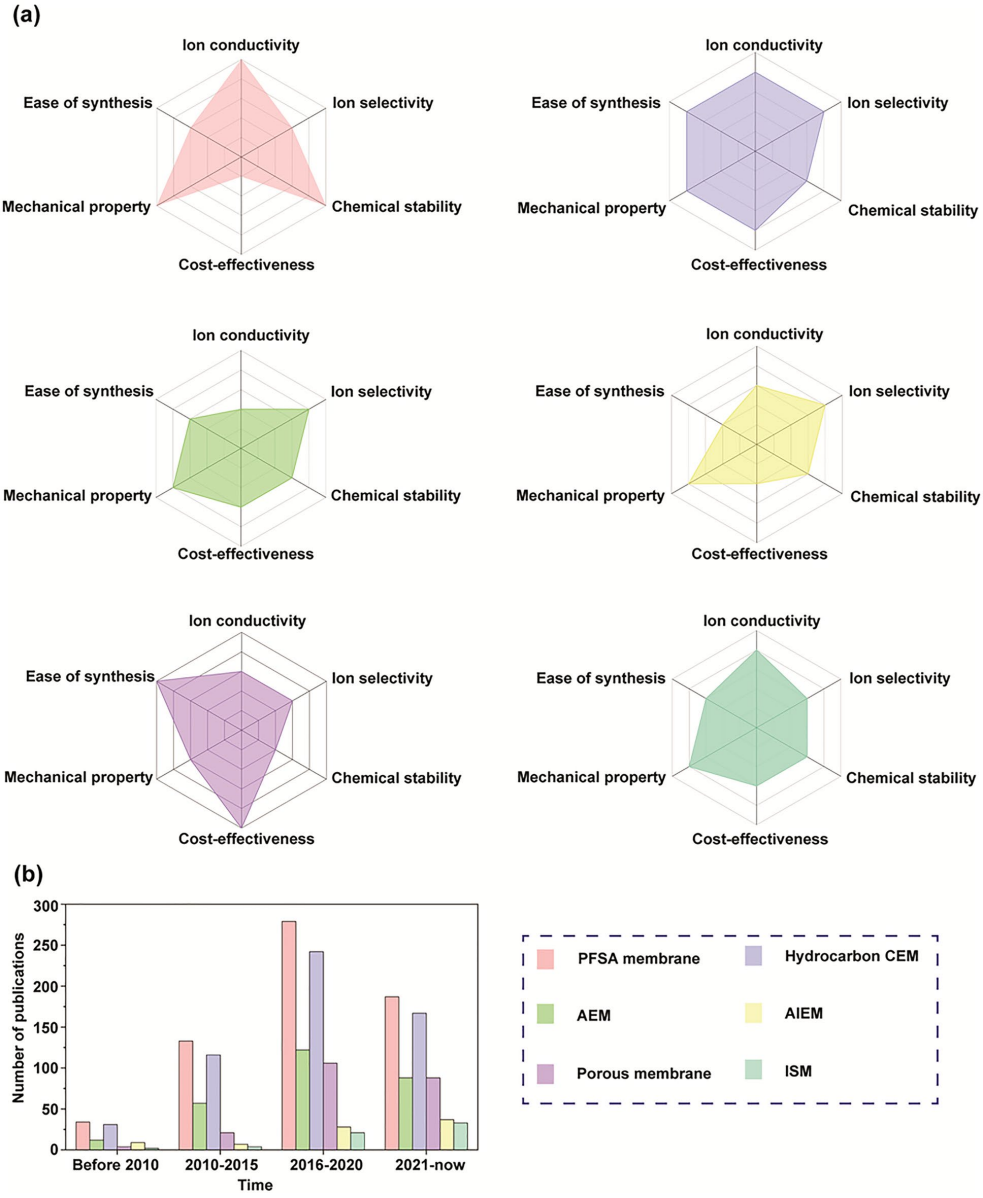
**a** Properties of different kinds of membranes. **b** Number of publications on different types of membranes for RFBs, the data are derived from the Web of Science database (accessed on 27 February 2025)

To summarise, the membrane is an important component that influences the lifespan, efficiency, stability, and cost of the VRFB. Major challenges for the commercial viability of VRFB include severe permeability of vanadium ion and the high cost of current commercial membranes. This study reviews polymer membranes pursuing high ion conductivity and low vanadium permeability to achieve high ion selectivity for VRFBs. Considerable research efforts combining material selection with different modification approaches have been reported to solve the trade-off between proton conductivity and vanadium permeability. Figure 13 illustrates the comparative performance, and the development of several membrane types used for VRFBs currently. Researchers have explored hydrocarbon ion exchange membranes as alternatives to mitigate the limitations associated with perfluorosulfonic acid membranes. AEMs with numerous positively charged functional groups in the backbone are more effective in limiting the vanadium ion crossover while CEMs have advantages in facilitating proton transportation because of the negatively charged functional groups. Porous membranes are the most cost-effective membranes due to the expansion in the material selection. However, the less effective ion selectivity via the size exclusion mechanism limited their application in VRFB applications. On the other hand, AIEMs combine the advantages of AEMs and CEMs, providing a promising approach to enhance the performance of VRFBs. Nevertheless, the overall performance of AIEMs during the operation of VRFBs has not been thoroughly investigated in the literature. Furthermore, the intricate nature of ionic clusters in the AIEMs complicates the synthesis process, which may potentially increase the cost of the membranes. ISMs represent an alternative approach using ion-solvation mechanism. While ISMs excel in providing high ion conductivity and adaptability, their drawbacks, including low ion selectivity, and poor mechanical and chemical strength highlight the need for further research into their application in VRFB applications. Compared to commercial membranes (Nafion membranes), the chemical stability of the hydrocarbon polymer membranes is relatively weak against the strong acidic and oxidising electrolyte in VRFB. Enhancing the chemical stability of hydrocarbon membranes has emerged as an urgent priority. To date, numerous material and modification techniques have been explored in attempts to improve the stability of membranes while maintaining high efficiency. Overall, carbon-based materials are widely used in membrane development because of their unique structures and mechanical and chemical properties. Their ability to form hybrid structures and provide uniform distribution of functional groups makes them ideal for creating membranes. Besides, organic materials and hybrid materials are highly promising candidates based on their versatility, exceptional properties, and ability to be tailored for specific applications. For modification techniques, the reviewed work indicated that introducing block groups via grafting, acid–base, and cross-linking structure could be promising approaches to enhance the oxidative stability of polymer membranes. Meanwhile, the pore-filling method has proven to be an effective approach for integrating the high conductivity of ionic groups and the excellent stability of porous membranes.

## Conclusion and Perspectives

VRFBs have emerged as a vital technology for large-scale energy storage systems due to their distinctive properties for storing energy, offering flexibility and scalability. The membranes have important roles in the VRFB applications, which determine the performance of battery cells. This membrane plays a dual role: separating the active species while facilitating the selective transport of supporting ions to ensure electrical charge balance during battery operation. The permeation of vanadium ions through the membrane during the operation process due to diffusion, migration, and osmosis pressure are critical issues for large-scale VRFB applications. Hence, this review provides a comprehensive overview of the ion transport mechanism that is crucial for exploring the appropriate membrane to attain high performance for VRFB applications. To reduce the vanadium ions permeability of the membrane, significant research efforts have been made involving material selection, novel hydrocarbon membrane synthesis, or membrane structure design. A statistical analysis of published literature, including material selection, methods, and strategies to enhance membrane performance for VRFB applications is presented in this review.

**Fig. 14:**
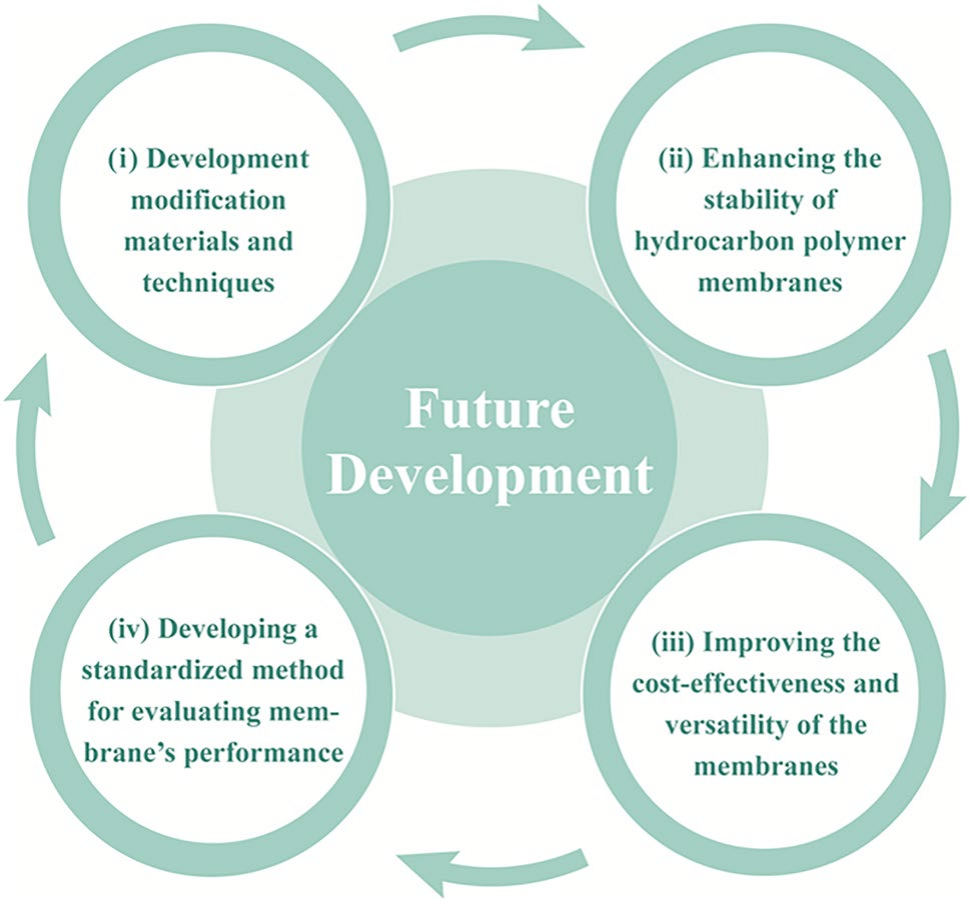
Perspectives for future research on membranes for VRFBs.

Figure 14 presents several perspectives to provide insights into future research on membranes for VRFBs. Although various materials and modification methods have been developed for VRFB membranes, the membranes developed currently are still far from meeting the requirements for practical applications. Reducing the permeability of active species crossover while keeping a reasonable ionic conductivity are key objectives for advancing membrane technology. In most cases, the fabrication of modified membranes focuses on reducing the permeation of vanadium ions via the blending/coating of organic/inorganic materials into the support membrane. These methods successfully reduce the permeability of active species across the membrane, hence reducing the capacity fade. However, an increase in electrical resistance has been observed that could be a critical issue limiting the ion conductivity of the membranes. Adding other materials into the membrane matrix also causes poor compatibility due to the mismatch in swelling ratio between the two components. Hence, further research on modification materials and methods is required to overcome the remaining drawbacks of membranes in VRFBs. Achieving these goals is urgently needed for enhancing battery efficiency and cycling performance in VRFB systems and then expanding the applications of VRFB in energy storage systems.

In addition, the sustainability and cost-effectiveness of the membrane are also critical requirements for membranes in VRFBs. Hydrocarbon polymer membranes are highly promising candidates for VRFB applications due to their high performance at a reasonable price. Nevertheless, the mechanical and chemical strength of these membranes is relatively weak compared to PFSA membranes, which leads to capacity fading resulting from vanadium ion crossover. Therefore, boosting the oxidative stability of hydrocarbon polymer membranes has become a critical priority for the future development of VRFBs. While the material offers significant cost advantages and environmental benefits, its chemical stability and ionic conductivity must be addressed through a combination of cross-linking, functionalisation, composite materials, and nanomaterials. These advancements will enhance the membrane’s durability, efficiency, making hydrocarbon-based membranes a viable option for the next generation of sustainable VRFB.

Further, it must be noted that a typical membrane is required for a specific battery or depending on the customer/application requirement. So far, using the same membrane for various RFB systems cannot achieve optimal performance because the different battery systems work in different conditions that require different expectations for the membrane. Therefore, modification strategies are required to enhance the versatility of the membrane, which is extremely important for future market development. In addition, the modified membrane should also be used in other fields of study such as water treatment, gas separation, fuel cells. Optimising the cost and versatility of the membrane is needed to minimise the cost of VRFB systems as well as the operating cost of VRFB applications.

Finally, standardised methods and testing protocols for evaluating membrane performance are needed to support the development of VRFBs. Despite numerous studies on membranes for VRFB employing similar characterisation methods, the procedures and the standard applied differ widely. A standardised testing protocol would ensure that membranes are evaluated under uniform conditions, facilitating direct comparison of performance data across different research studies. Consistent testing methods lead to reliable, reproducible results that are essential for advancing technology. This standardised approach will promote the commercialisation and extensive adoption of VRFBs, establishing a foundation for further research and industry innovation.
